# Study on Evaluation of Fruit Aroma of Plum Variety Resources Based on Headspace Solid-Phase Microextraction Combined with Gas Chromatography–Mass Spectrometry

**DOI:** 10.3390/foods13213515

**Published:** 2024-11-03

**Authors:** Hailong Sun, Xiaofeng Lu, Yang Wang, Jing Li, Shuo Liu

**Affiliations:** 1 Institute of Pomology of CAAS, Xingcheng 125100, China; sunhailong@caas.cn (H.S.); wangyang@caas.cn (Y.W.); lijing01@caas.cn (J.L.); 2 Key Laboratory of Horticultural Crops Germplasm Resources Utilization, Ministry of Agriculture and Rural Afffairs, Xingcheng 125100, China; 3 Liaoning Institute of Pomology, Yingkou 115009, China

**Keywords:** plum, aroma components, maturity period, HS-SPME-GC-MS, aroma character

## Abstract

To explore the characteristics of and variations in aroma components across different plum varieties and maturity stages, this study employed headspace solid-phase microextraction combined with gas chromatography–mass spectrometry (HS-SPME-GC-MS). This method was used to systematically analyze the aroma components of 12 early-maturing, 15 medium-maturing, and 11 late-maturing plum varieties. The variations in volatile components among these three germplasm types were then compared using multivariate statistical methods. The examination revealed that 138 aromatic components were meticulously identified and quantified, such as 26 aldehydes, 63 esters, 13 ketones, 30 alcohols, and six other compounds. Thirteen main aroma compounds including acetic acid hexyl ester, (Z)-3-hexen-1-ol acetate, hexanal, 1-hexanol, 3-hexenal, butanoic acid butyl ester, (E)-2-hexen-1-ol, hexanoic acid butyl ester, propanoic acid butyl ester, (E)-2-hexenal, L-.alpha.-terpineol, (Z)-2-hexen-1-ol acetate, and 1-butanol were considered dominant. The orthogonal partial least squares discriminant analysis (OPLS-DA) combined with variable importance projection (VIP) results showed that 24 differential aroma compounds were screened out from 38 varieties of plum fruits based on their differences in aroma components, which can be used to distinguish plum fruits at different ripening times. Twenty-four aroma-contributing compounds were identified based on their odor activity values (OAVs). Among these, 14 key aroma components with OAVs ≥ 10 were highlighted. In summary, the aroma compounds of early- and late-maturing plum germplasm exhibited rich diversity, with significant differences in aroma components between plums of varying maturity and germplasm. These differences can serve as indicators for identifying different plum germplasm.

## 1. Introduction

Plum belongs to the *Prunus* genus in the Rosaceae family. It has a long cultivation history in China and is also an economically important fruit worldwide due to its bright color and rich taste. With the shift in agricultural industry structure and the decline in the benefits of traditional dominant fruits, plums have become a key driver in advancing the fruit industry and promoting rural revitalization. This is due to their strong adaptability, early fruiting, rapid yield, quick return on investment, ease of management, and economic, social, and ecological benefits [1]. According to FAO (2022) statistics, the cultivation area of plum in China is 1.978 million hectares, with a yield of 6.63 million tons [2]. Currently, there are many types of plums available in the market, and the harvesting period in Northeast China can extend from late June to mid-September. While consumers have more choices, aroma, an important indicator of fruit flavor quality, has become one of the important factors, attracting consumers and enhancing their purchasing power [3]. Therefore, fruit aroma breeding has gradually become a hot research field, and exploring the changes in the aroma components of different mature plum varieties is crucial in clarifying the aromatic compound structure of plum fruits and screening specific plum germplasm research. During the fruit ripening process, aromatic compounds impart a unique odor to plants, affecting the quality of fruit aroma. They also have important effects on pollination and plant defense [4,5]. The aroma of fruits is relatively complex, and current research suggests that the types and contents of their intrinsic volatile components are the main reasons for presenting different aroma characteristics [6,7]. Jiang et al. pointed out an investigation into the volatile compounds and aroma characteristics of litchi fruits from six distinct germplasms, categorized into special-early-maturing and early-maturing types. Their findings revealed that the alcohols, aldehyde ketones, and lipids were the main volatile components of the special-early germplasm. In contrast, the early germplasm was characterized by a different set of main volatile compounds, with terpenoids, including both monoterpenes and sesquiterpene, as well as alcohols, being the most significant [8]. At present, more than 100 aroma components have been detected in plum fruit, with the primary types being alcohols, esters, aldehydes, ketones, terpenes, and phenols, among which alcohols, esters, and aldehydes have the highest content [9,10]. Williams et al. revealed that compounds such as nonanal, 1-hexanol, benzaldehyde, γ-octolactone, and γ-decanoide account for a significant proportion of the aroma components in plum fruit [11]. Zhang et al. found that benzene, isoborneol, (E,Z)-2,6-nonadienal, and benzyl tiglate could serve as markers to distinguish the primary substances in the ‘Kongxin’ plum [12]. Among the current plum cultivation varieties, most are medium to late maturing, while there is a relative scarcity of high-quality early-maturing varieties. The evaluation of fruit quality mainly focuses on indicators such as fruit size, sugar and acid components, and anthocyanin content [13]. Researchers are primarily focused on the presence or absence of aroma during the breeding process, with limited investigation into the types, contents, and compositions of aroma substances [14]. This study selected 38 plum varieties, mainly cultivated in Liaoning, Jilin, and Heilongjiang provinces, to compare, analyze, and identify the content of mature flesh aroma compounds in early- and mid–late-maturing plum germplasm. By determining the composition of aroma components at different maturity stages, the research aims to provide new insights for the assisted identification and breeding of plum germplasm.

## 2. Materials and Methods

### 2.1. Fruit and Reagent Materials

Thirty-eight representative and superior plum varieties with different maturity periods were selected from the experimental base at the Institute of Pomology, CAAS (Xingcheng, Liaoning Province, China) between July and September. These included 12 early-maturing varieties, 15 medium-maturing varieties, and 11 late-maturing varieties. The cultivar number, name, classification, and maturity are listed in Table 1. The plums selected for this research were consistent in both size and color, ensuring uniformity across the samples. Additionally, they were carefully inspected to ensure the absence of mechanical damage, as well as any signs of diseases or pests. For the experimental procedures, ten fruits were combined to form a single replicate. The method involved three biological replicates to ensure the reliability and accuracy of the results obtained. Following the harvest, the fresh fruits were packed and promptly delivered to the laboratory for immediate analysis of aroma components. 3-Octanol (>98%), designated as the internal reference, was supplied by Shanghai Macklin Biochemical Co., Ltd. (Shanghai, China). A standard solution was formulated using a 10% methyl alcohol mixture. For this purpose, methyl alcohol of HPLC grade was procured from Fisher Scientific (Pittsburgh, PA, USA). Purified water was provided by the Milli-Q 7003 Pure & Ultrapure Water Purification System (Merck KGaA, Darmstadt, Germany). Sodium chloride (NaCl), employed across each of the experiments, was sourced from Agela Technologies (Newark, DE, USA).

### 2.2. Sample Preparation

The fruit flesh was cut using a knife after the core was removed (IKA company, Germany) and combined with NaCl (5:1, m/m) before being homogenized with a homogenizer. Subsequently, an 8 g portion of the mixture was carefully measured out and subsequently placed within a 20 mL SPME vial for further analysis. Next, a 20 µL 3-octanol standard solution (8.75 mg·L^−1^) was precisely dispensed within the vial. To ensure the vial’s contents were securely contained, it was then promptly closed off utilizing a screw cap equipped with a polytetrafluoroethylene/silicone septum.

### 2.3. GC-MS Analysis

Solid-phase microextraction conditions were as follows: 50/30 μM DVB/CAR/PDMS extraction fiber was used for sample extraction. The extraction head of the solid-phase microextractor was inserted into the sample bottle through the rubber pad on the bottle cap, and the fiber head was pushed out. Care was taken to ensure that the extraction head did not touch the fruit particles. Adsorption occurred at a constant temperature of 60 °C for 30 min, after which the fiber head was pulled back, and the extraction head was removed from the sample bottle. The extraction head was then quickly inserted into the gas chromatograph, the fiber head was pushed out, and the instrument was started to collect data. Analysis was conducted at 240 °C for 3 min, after which the fiber head was pulled back, and the extraction head was removed.

GC conditions were as follows: An HP-INNOWAX capillary column was installed in the chromatograph (60 m × 0.25 mm × 0.25 µm), which was procured from Agilent Technologies, Santa Clara, CA, USA. The column was initially programmed to a temperature of 50 °C, which was sustained for a duration of 1 min to stabilize the conditions. During subsequent operations, the temperature was raised progressively by 10 °C per minute until it reached 250 °C and a connection port temperature of 280 °C. Helium (99.999% purity), the main carrier gas during the study, was used and it was necessary to ensure that the flow rate was meticulously maintained at 1.0 mL/min throughout the process. The detector temperature, ion source temperature, and temperature of the quadrupoles rod were established at 270 °C, 230 °C, and 150 °C.

Mass spectrometry relies heavily on the electron ionization (EI) mode, which requires that the ionization energy be set at a standard of 70 eV and that the operation be conducted at 230 °C. The mass spectrum was acquired through the use of the full scan mode, which encompassed an extensive scanning range extending from *m*/*z* 50 to 500. Supported by GC-MS analysis, total ion chromatograms were yielded and mapped in the study, utilizing the NIST 17 s database as a reference standard to identify and characterize the metabolites in the samples. Volatile compounds exhibiting a match of 90% or higher were retained for further analysis. Subsequently, the corresponding CAS registry numbers and retention times (RTs) of these compounds were professionally searched and meticulously queried. Each piece of data was derived from reliable results of experiments conducted in triplicate. The quantitative analysis of volatile components in the sample was performed using the internal standard method, with 3-octanol serving as the internal standard. A single standard of 3-octanol was prepared as the internal standard solution and added to the sample.

### 2.4. Data Processing and Statistical Analysis

Quantitative analysis of aroma components involved calculating their concentrations by assessing the peak area and content of detected aroma components with those of internal standards. Calculation of the aroma intensity value (OAV) was performed where the OAV represents the proportion of a particular aroma component’s concentration relative to its established threshold level [15].

Data analysis and graphical presentation were conducted using Excel 2010 software, which facilitated the organization and visualization of the results. SIMCA version 14.1, provided by Umetrics in Umea, Sweden, was utilized for conducting OPLS-DA and VIP. Correlation analysis was meticulously carried out through the application of SPSS 20.0 software (International Business Machines Corporation, Armonk, NY, USA).

## 3. Results and Analysis

### 3.1. Analysis of Differences in 38 Plum Resources of Fruits’ Aromatic Components

**Figure 1:**
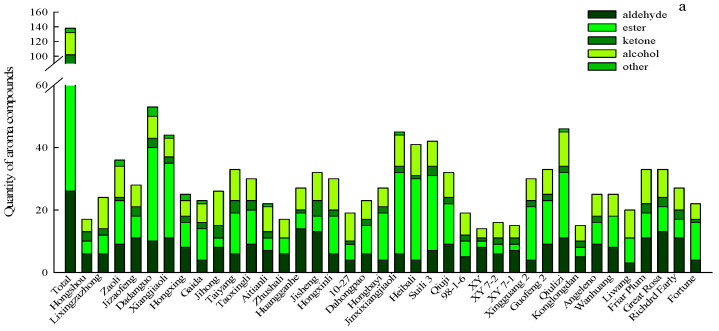

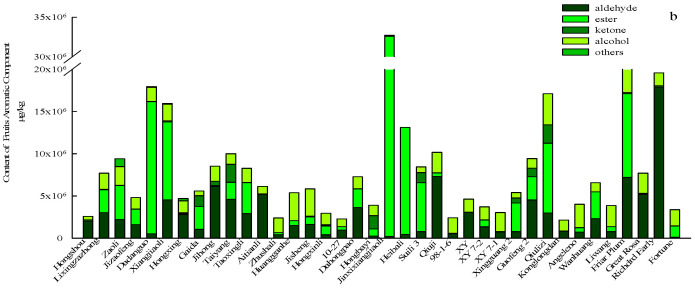
Aromatic components of the types and contents in 38 plum germplasm resources. (**a**) shows the number of aroma compounds among different plum germplasm resources, (**b**) shows the contents of total volatile compounds in 38 plum germplasm resources.

Previous findings indicated that aldehydes and esters constituted the most abundant classes of compounds present in plum fruits [16]. This study yielded comparable findings. Based on the variation in the primary functional groups of the compounds, successful quantification was completed of 138 identified aroma components, comprising 26 aldehydes, 63 esters, 13 ketones, 30 alcohols, and six other compounds in 38 plum germplasm resources, as shown in Figure 1a.

Notably, the number of aroma compounds varied significantly among different plum germplasm resources, ranging from 14 to 53. Dadanguo exhibited the highest number of aroma components, with 53 types. This was followed by Qiulizi and Jinxixiangjiaoli, with 46 and 45 types, respectively. XY had the lowest count, with only 14 aroma components. Among the 38 tested plum resources, Huangganhe plum had the highest number of aldehyde substances in its fruit, containing 14 species; Dadanguo plum had the highest number of esters, with 30 types; Jisheng plum had the highest content of ketones, containing 14 species; and Qiulizi and Friar plums had the highest alcohol content, both at 11 species. In XY, aldehyde aroma components constituted the largest proportion of total aroma components, at 57.14%. In Heibali, ester aroma components made up the highest proportion, accounting for 63.41%. Jisheng had the highest proportion of ketone aroma components at 15.63%. The highest proportion of alcohol aroma components was found in fruits from 10 to 27, at 47.37%. Among 38 plum varieties, the number of aldehydes ranged from 3 to 13, esters from 3 to 30, and alcohols from 3 to 11. Ketones were relatively scarce, ranging from 0 to 5.

The results indicate that esters, aldehydes, and alcohols were the leading contributors to the aroma profile, collectively comprising over 78.00% of the total content. Only a small fraction of the resources was primarily composed of ketones. Among the varieties, Jinxixiangjiaoli had the highest ester content, measuring 323,467.43 μg·kg^−1^, which accounted for 98.89% of its total aroma components. This was followed by Dadanguo and Heibali, with ester contents of 156,175.28 μg·kg^−1^ and 126,476.45 μg·kg^−1^, respectively. XY had the lowest ester content, at 145.75 μg·kg−^1^. The aldehyde content in the 38 varieties ranged from 2226.34 to 178,856.08 μg·kg−^1^, with Richard Early having the highest aldehyde content, representing 91.36% of its total aroma components. XY 7-1 had the highest alcohol content, accounting for 74.19% of its total. The aroma components of Taiyang fruit were predominantly ketones, making up 46.17% of its total aroma components (Figure 1b).

Among the aroma components in 38 plum varieties, 13 main compounds were dominant, with the highest contents being as follows: acetic acid hexyl ester, (Z)-3-hexen-1-ol acetate, hexanal, 1-hexanol, 3-hexenal, butanoic acid butyl ester, (E)-2-hexen-1-ol, hexanoic acid butyl ester, propanoic acid butyl ester, (E)-2-hexenal, L-alpha-terpineol, (Z)-2-hexen-1-ol acetate, and 1-butanol (Table 2). However, for different varieties of aroma substances, it is not only the type that shows variability but also the content. Acetic acid hexyl ester had the highest content in the aroma components of Dadanguo, Jinxixiangjiaoli, and Heibali. In Dadanguo, the second most abundant compound was (Z)-3-hexen-1-ol acetate, followed by butanoic acid butyl ester. In Jinxixiangjiaoli and Heibali, the third most abundant compound was hexanoic acid butyl ester, but the second most abundant was different: (Z)-3-hexen-1-ol acetate in Jinxixiangjiaoli and 1-hexanol in Heibali. For the fruits of Zaoli, Taiyang, Jisheng, Hongxinli, Suili 3, and Liwang, (Z)-3-hexen-1-ol acetate and 1-hexanol were the substances with the highest aroma content, respectively. In fruits such as Gaida, Friar Plum, and Fortune, (Z)-3-hexen-1-ol acetate emerged as the predominant aroma compound. Additionally, (E)-2-hexenal, (Z)-2-hexen-1-ol acetate, (E)-2-hexen-1-ol, 1-hexanol, acetic acid hexyl ester, and hexanoic acid butyl ester were significant. For the Jihong variety of fruits, the order of substances with the highest content was hexanal > 1-hexanol > (E)-2-hexen-1-ol. For Richard Early fruit, the order was hexanal > (E)-2-hexen-1-ol > (E)-2-hexenal. The highest contents of fruit aroma substances were those of formic acid and acetic acid. In Lixingzazhong, Jizaofeng, XY, XY7-1, XY7-2, and Great Rosa, high levels of 1-hexanol and (E)-2-hexen-1-ol were observed. Conversely, Xiangjiaoli and Hongxing fruits exhibited significant amounts of 3-hexenal and (Z)-3-hexen-1-ol acetate. In addition, the order in Richard Early fruit was (E)-2-Hexen-1-ol > Hexanal > (Z)-2-Hexen-1-ol acetate. Aroma components, not only in type but also in content, varied to some extent among the varieties, showing that further analysis of the aroma components in the 38 varieties of plums had certain significance.

### 3.2. Correlation Analysis Between Total Content of Aromatic Components and Each Aroma Substance

**Figure 2:**
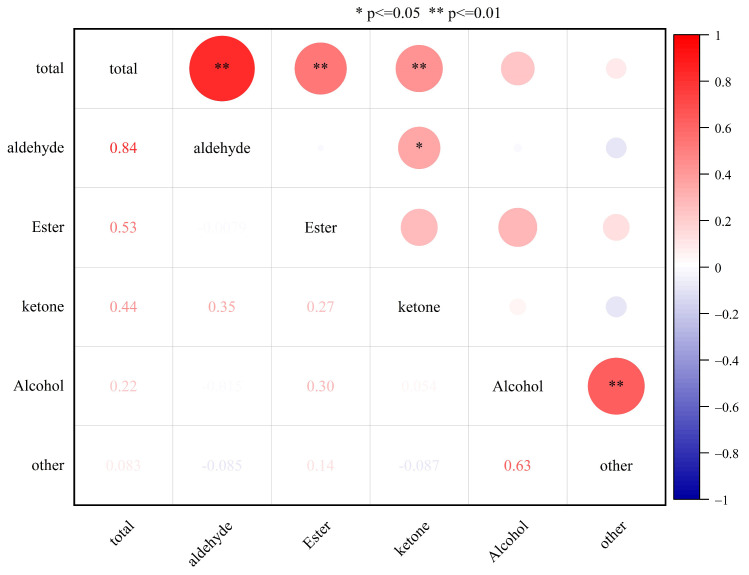
Correlation analysis conducted between the total content of aromatic components and each aroma substance. Significance levels are denoted with an asterisk (* for *p* < =0.05, ** for *p* <= 0.01). The depth of color in the figure reflects the strength of the Pearson correlation coefficient.

The positive correlation between the overall quantity of aromatic components and the contents of aldehydes, esters, and ketones in 38 plum varieties was highly significant, as shown in Figure 2. This indicates that higher total aromatic content in plums is associated with higher levels of aldehydes, esters, and ketones. Additionally, the positive correlation between alcohol content and other compounds was significant. However, the correlation between other types of substances was not significant, suggesting no necessary correlation among the different types of aromatic components in the 38 plum germplasm resources.

### 3.3. Analysis of Key Aroma Components in 38 Plum Fruits at Different Maturation Stages Based on OPLS-DA

**Figure 3:**
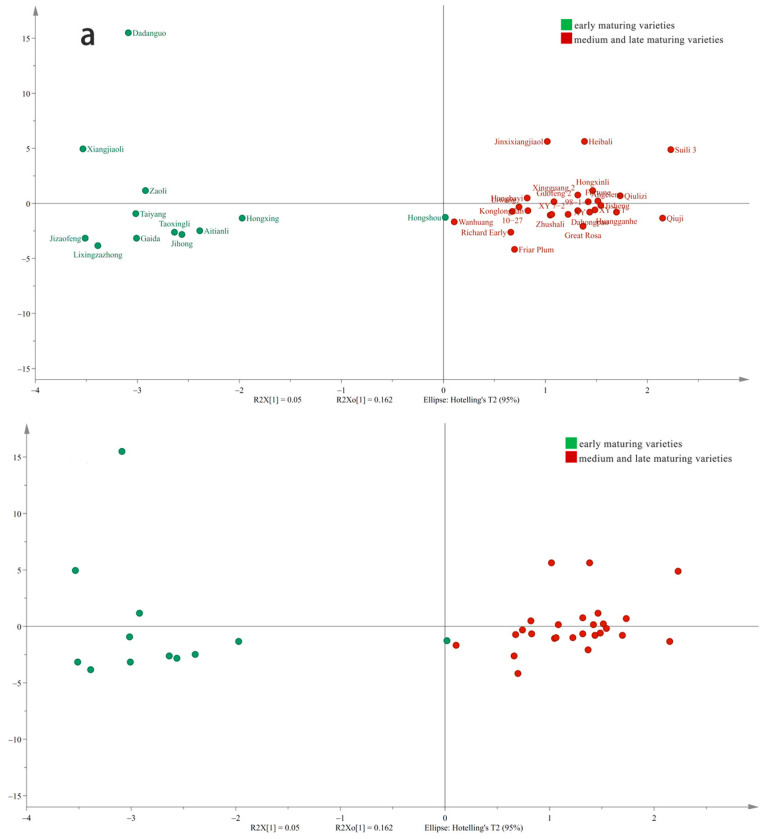

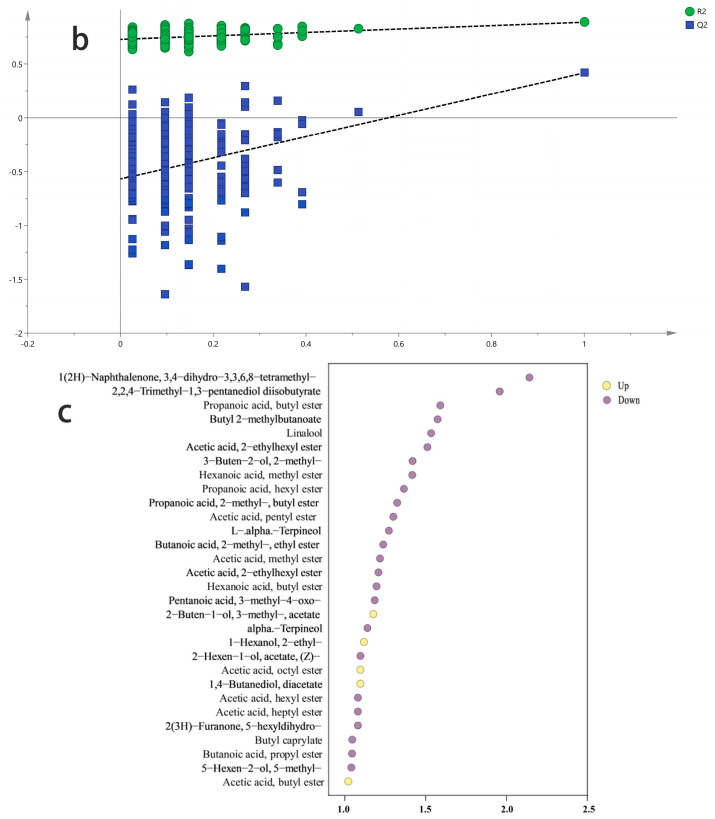
Key aroma components in 38 plum fruits at different maturation stages based on OPLS-DA. Results of OPLS-DA (**a**), model cross-validation result (**b**), and VIP values (**c**) of aromatic components in 38 plum resources. The bars displayed in red represent the differential volatile compounds that were identified under the criterion where VIP > 1.

As a typical multivariate calibration technique, the complexity of OPLS-DA is well recognized in the academic community [17,18]. By focusing on maximizing the separation between predefined groups, OPLS-DA effectively aids in the detection and analysis of metabolic variations. To examine the variations in aroma compounds among different plum varieties, 138 aroma compounds served as the dependent variable. Meanwhile, these aroma compounds’ density across different mature plum varieties was used as the independent variable (Figure 3). OPLS-DA was used to divide the 38 plum varieties into two groups. The first group consisted of early-maturing varieties, distributed in the first and fourth quadrants; the second group was composed of medium- and late-maturing varieties, mostly distributed in the second and third quadrant. The distribution of each variety was relatively uniform. The repeatability of the aroma at the same maturity stage was good, suggesting that there are notable differences at various maturity stages (Figure 3a). Model validation demonstrated that after 200 permutation tests, the permutation test model yielded R2X = 0.364, R2Y = 0.887, and Q2 = 0.417 (Figure 3b), underscoring the model’s significance. The point where the Q2 regression line intersects the vertical axis, which was observed to be below 0, signifies that the discriminant model possesses strong explanatory power. This finding also indicates that the model did not suffer from overfitting. Consequently, the validation of the model can be considered to be effective.

In the context of OPLS-DA, the VIP score reflects the extent to which differences in specific metabolites contribute to the classification and identification of sample groups within the model. Generally, metabolites are deemed significantly different if they exhibit VIP > 1 and *p* < 0.05 [19]. The OPLS-DA model was used as the outcome control criterion; 24 aroma components were identified as differential, characterized by VIP > 1 and *p* < 0.05 (Figure 3c). Among these components, 13 were esters, seven were alcohols, two were aldehydes, one was a ketone, and one was classified as another type of compound. The model contained seven up-regulated substances: 2,2,4-trimethyl-1,3-pentanediol diisobutyrate, linalool, benzoic acid 2-ethylhexyl ester, propanoic acid butyl ester, butyl 2-methylbutanoate, acetic acid methyl ester, and (E)-2-hexenal. This indicates that the levels of these aroma substances are significantly higher in mid- to late-maturing plum germplasm compared to early-maturing ones. Additionally, there were 17 down-regulated substances: 3,4-dihydro-3,3,6,8-tetramethyl-1(2H)-naphthalenone, 2-methyl-butanoic acid ethyl ester, hexanoic acid methyl ester, 2-methyl-propanoic acid butyl ester, (Z)-2-hexen-1-ol acetate, propanoic acid hexyl ester, acetic acid 2-ethylhexyl ester, 3-methyl-2-buten-1-ol acetate, (Z)-butanoic acid 3-hexenyl ester, benzaldehyde, 2-methyl-3-buten-2-ol, L-alpha-terpineol, alpha-terpineol, 5-methyl-5-hexen-2-ol, (E)-4-hexen-1-ol, 2-ethyl-1-hexanol, and 3-methyl-4-oxo-pentanoic acid. This shows that the levels of these aroma substances are significantly lower in mid- and late-maturing plum germplasm compared to early-maturing ones. These unique aroma substances with high abundance of specific compounds can be used as one of the markers to distinguish plum germplasm of different maturity stages.

### 3.4. Principal Component Analysis of Aroma Components in 38 Plum Germplasm Resources

According to Table 3, 138 aroma components of plums from 38 varieties were classified into 16 categories (based on the principle of eigenvalues greater than 3), which captured 81.66% of the total variance, thereby encompassing the majority of the information in the original data. Based on the reported compound aroma thresholds in the existing literature, the aroma values of various aroma components in each variety were calculated, thereby preliminarily determining the characteristic aroma components in the key aroma substances of each variety.

Focusing on 71 key aroma components from 38 plum resources, 24 specific aroma components were identified among these key aromas, indicating a significant contribution to aroma characteristics (OAV > 1), as shown in Table 4. Additionally, there were 14 substances with a concentration exceeding 10, and these are considered specific aroma components of key aroma components (Table 5). Among the 38 plum varieties, four specific aroma components, 2-Hexenal, (Z)-3-hexen-1-ol acetate, Academic acid butyl ester, and (E)-2-Hexenal, were present in most plum-specific aroma components. Jihong, Taiyang, and Aitianli fruit had a more prominent OAV for the specific aroma substance 2-Hexenal, with a strong leaf fragrance. The OAV of (E)-2-Hexenal in Gaida was much higher than other varieties, and the pleasant green leaf fragrance and fruit aroma in the fruit were more intense. (Z)-3-hexen-1-ol acetate in Zaoli, Dadanguo, and Xiangjiaoli fruits resulted in a strong banana aroma in their fruit aroma. The strong fruity taste in Jinxiangjiaoli comes from the high OAVs of acetic acid butyl ester, which contribute significantly to its aroma characteristics. 

In terms of the number of specific aroma components, Dadanguo and Heibali contained the most, with 10 types. Among them, the OAV ranking of the aroma components in Dadanguo fruits was (Z)-3-hexen-1-ol acetate > 2-hexenal > acetic acid pentyl ester > 2-methylbutanoate Butyl > hexanoic acid methyl ester > 1-butanol > 2-methyl-1-butanol > (E)-2-hexenal > 1-Octanol > butanoic acid, mainly presenting with a banana aroma, leaf flavor, and fruit sweet taste. And 1-Octanol is a unique aroma component that is not found in other varieties of plum fruits, with a unique lemon flavor. The main aromatic substance in Heibali was acetic acid butyl ester, (Z)-3-hexen-1-ol acetate, 1-Cyclohexene-2-hexenal and 2,6,6-trimethyl -1-carboxaldehyde; the fruit aroma mainly presents as a strong banana aroma, fruit flavor, leaf aroma, and tropical fruit aroma.

The five varieties of plum XY 7-2, Konglongdan, Great Rosa, Richard Early, and Fortune contained the least specific aroma components, all of which had two and did not contain unique aroma components. Among them, the four varieties of XY 7-2, Konglongdan, Great Rosa, and Richard Early had the same specific aroma components, which were (E)-2-hexenal and 2-hexenal, with a fruity and pleasant fruit aroma. The specific aroma components of Fortune were acetic acid butyl ester and (Z)-3-hexen-1-ol acetate, which trigger a strong banana aroma and fruity taste. Xiangjiaoli contained the most unique aroma compounds, ethyl acetate, formal acid, and butyl ester, which give its fruit a unique mume-like aroma and sweet fruity taste. The unique aroma component of Hongxing was octanal, with an OAV of 120.06, which has a strong aroma similar to rose and orange peel. Gaida’s unique pentanoic acid ethyl ester components give its fruit an apple-like fruity aroma. Jizaofeng’s unique 1-octen-3-one ingredient gives its fruit a herbal, mushroom, and earthy aroma. The unique aroma components in Lixingzazhong fruit are (Z)-3,7-dimethyl-2,6-Octadien-1-ol, which impart pleasant rose and orange blossom aromas in the fruit, with a hint of a lemon-like fruity aroma. The content of specific aroma components in other varieties ranges from three to nine, and none of them have unique aroma component substances.

**Figure 4:**
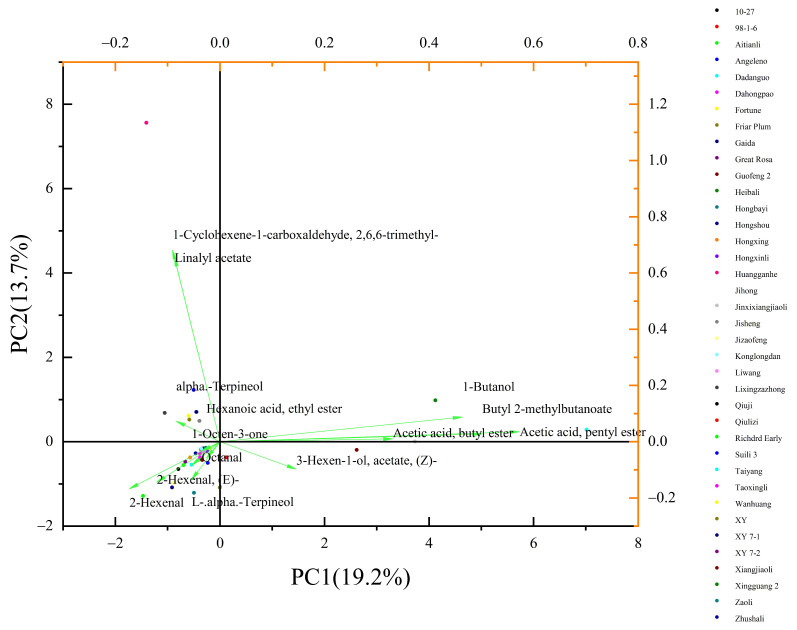
PCA of 14 extremely key aroma components in 38 plum fruits.

According to the principal component analysis of extremely key aroma substance characteristic values, the 38 tested materials were divided into five categories (Figure 4). Among them, Hongshou was distributed separately in the second quadrant and the main contributors were Linalyl acetate and 1-Cyclohexene-1-carboxyaldehyde, 2,6,6-trimethyl. Dadanguo, Jinxixiangjiaoli, and Heibali were distributed near the x-axis in the first quadrant, with 1-butanol, butyl 2-methylbutanoate, acetic acid, butyl ester, acetic acid, and pentyl ester playing the main contributing role. Xiangjiaoli was distributed in the fourth quadrant, and the main contributor is 3-Hexen-1-ol, acetate, (Z)-. Lixingzazhong, Angeleno, XY 7-1, Wanhuang, Friar Plum, XY, and Jisheng were distributed in the second quadrant, and alpha.-terpineol, hexanoic acid, ethyl ester, and 1-octen-3-one play a major role. The remaining varieties were distributed in the third quadrant and had similar main aroma components, with Octanal, 2-Hexnal, and (E)-L. alpha.-terpineol 2-Hexnal playing a major role.

## 4. Discussion

HS-SPME-GC-MS technology is recognized as a leading method that can be used for extracting and determining aroma substances both domestically and internationally. It utilizes the high temperature of the gas chromatography injection port to analyze the adsorbed components from the stationary phase through chromatography [27]. Li et al. used this technology to detect the total number of VOC species, 148, at different stages in the maturity of passion fruit, which were mainly esters (approximately 30%), terpenes (13%), ketones (11%), aldehydes, alkanes, alcohols, amides, phenols, etc. [28]. In another study, GC-MS analysis identified 48 compounds across three kiwifruit resources, encompassing aldehydes (16), alcohols (11), esters (16), and other compounds (5) [29]. During the course of this study, the technique was fully utilized for the professional identification and precise quantification of 38 plum germplasm resources, resulting in the detection of 138 aromatic components in total, encompassing 26 aldehydes, 63 esters, 13 ketones, 30 alcohols, and six other compounds. The aroma compounds contained in different types of fruits vary somewhat, both in type and in content. Only 30 volatile compounds were detected in Wushancuili plum, encompassing four alcohols, 10 aldehydes, four esters, four acids, three ketones, one alkane, one aromatic compound, two terpenes, and one heterocyclic compound. *Prunus salicina*, along with its hybrids, exhibited a considerably greater diversity and higher concentration of volatile compounds compared to *Prunus domestica* and various other Chinese wild species. While aldehydes were identified as the predominant volatile compounds in both *P. domestica* and *Prunus spinosa*, it was esters that dominated the volatile profiles in *P. salicina* and its hybrids, as well as in *Prunus ussuriensis* fruits [30]. Most researchers believe that the aroma components of plum are mainly aldehydes, esters, alcohols, and ketones, which affect the flavor of the fruit. In this study, we found that 38 varieties of plums contain a large amount of aldehydes, esters, alcohols, and ketones, which are extremely abundant and the main aroma components in plum fruit flesh, with aldehydes and esters accounting for the majority. The positive correlation between the overall quantity of aromatic components and the contents of aldehydes, esters, and ketones was highly significant; this result is similar to Xu et al.’s study on the changes in aroma content during the shelf life of pears with different flesh types [31].

Previous studies have mainly focused on the differences in aroma compounds among different germplasm types, and there have been no reports on the characteristics at different maturity stages. In this study, we found that the cumulative content and quantity of aroma substances in the middle- and late-maturing groups were generally higher than those in the early-maturing group, with a stronger aroma of the middle- and late-maturing plum fruit. By using OPLS-DA, different plum germplasms can be clearly distinguished and clustered into two categories based on maturity. This indicates that the aroma substances of the same maturity germplasm have high similarity but are not entirely the same; the result is similar to the research on litchi fruits of six special-early-maturing germplasm, as well as early-maturing germplasm [8]. In addition, the VIP value clearly displayed the significantly different aroma substances in various germplasm samples, indicating that these substances can be used as characteristic indicators to distinguish between the 38 plum varieties based on their maturation stages, namely early maturing, and medium and late maturing.

A fruit can synthesize hundreds of volatile compounds, but only a small fraction of substances that reach the flavor threshold play a major role in the aroma, which is the characteristic aroma of the fruit [32]. The aroma of the fruit is influenced by multiple compounds, and a higher content does not necessarily mean a greater contribution to the aroma. Only certain characteristic compounds have a significant impact on the fruit’s aroma [33,34]. According to the differences in sensory effects, fruit aromas can be classified into fruity, green, spicy, woody, aldehyde, and others [35]. Esters endow fruits with floral and fruity aromas, belonging to fruity compounds. Aldehydes and alcohols have a green aroma of green grass and leaves, while terpenes are the main components of floral fragrances [36]. From the types and aroma values of characteristic aromas, ‘Hongshou’, Aitianli, 98-1-6, XY, XY 7-2, XY 7-1, Konglongdan, Great Rosa, and Richard Early are varieties mainly composed of aldehydes, belonging to the aldehyde fragrance type. Dadanguo, Xiangjiaoli, Hongxing, Taiyang, Taoxinli, Zhushali, Jisheng, Hongxinli, 10-27, Dahongpao, Hongbayi, Jinxixiangjiaoli, Suili 3, Qiuji, Xingguang 2, Guofeng 2, Qiulizi, Angeleno, and Liwang are of the aldehyde alcohol mixed type. Lixingzazhong, Zaoli, Gaida, Jihong, Huangganhe, Heibali, Wanhuang, and Friar Plum are mainly composed of aldehydes, esters, and alcohols, forming a mixed aroma of aldehyde, ester, and alcohol. Jizaofeng is mainly composed of aldehydes, esters, and ketones, with a mixed aroma of aldehydes, esters, and ketones. Fortune belongs to the ester fragrance type. (E)-2-hexenal (green, aldehyde, fruity, spicy, and fatty), and 2-hexenal (leafy), butyl acetate (fruity), and (Z)-3-hexen-1-ol acetate (strong banana) are common aroma compounds and primary components in most plum varieties. Additionally, each variety has its own unique aromas, such as octanal (extremely diluted with aromas like rose and orange peel) in Hongxing fruit, acetic acid pentyl ester (fruity and sweet) in Dadanguo fruit, and 1-octen-3-one (herbs, mushrooms, soil) in Jizaofeng fruit. These findings highlight the diversity of fruit aroma components, similar to research on the diversity of peach germplasm aromas [37]. They are all excellent plum resources rich in unique aromas, and are good parents for aroma breeding. Hybridizing with plum resources containing unique aroma components can improve the efficiency of aroma breeding. The excavation of these unique aroma plum resources can provide an important reference for the breeding of new plum varieties (lines) rich in various aroma types, and can also provide reference for the regulation of aroma substances such as octanal and acetic acid pentyl ester.

## 5. Conclusions

This study systematically analyzed the aroma components of 12 early-maturing, 15 medium-maturing, and 11 late-maturing plum varieties, comparing the differences among these three types of germplasm. The results identified and quantified a total of 138 aromatic components, including 26 aldehydes, 63 esters, 13 ketones, 30 alcohols, and six other compounds in 38 plum germplasm resources. Thirteen main aroma compounds were dominant. The OPLS-DA combined with VIP prediction results revealed 24 differential aroma compounds that distinguish plum fruits at different ripening times, serving as characteristic volatile compounds. Based on their aroma activity value (OAV), 24 compounds contributing to the aroma were screened, resulting in 14 key aroma components. In summary, the aroma compounds of early- and late-maturing plum germplasm show rich diversity, with significant differences in aroma components between plum fruits of different maturities and germplasm. These differences can be used as indicators for identifying various plum germplasms.

## Figures and Tables

**Table 1:** Plum cultivar number, name, classification, and maturity of plum cultivars used in this work.

Cultivar NO.	Cultivar Name	Maturity	Cultivar NO.	Cultivar Name	Maturity
1	Hongshou	Early	20	Jinxixiangjiaoli	Medium
2	Lixingzazhong	Early	21	Heibali	Medium
3	Zaoli	Early	22	Suili 3	Medium
4	Jizaofeng	Early	23	Qiuji	Medium
5	Dadanguo	Early	24	98-1-6	Medium
6	Xiangjiaoli	Early	25	XY	Medium
7	Hongxing	Early	26	XY 7-2	Medium
8	Gaida	Early	27	XY 7-1	Medium
9	Jihong	Early	28	Xingguang 2	Late
10	Taiyang	Early	29	Guofeng 2	Late
11	Taoxingli	Early	30	Qiulizi	Late
12	Aitianli	Early	31	Konglongdan	Late
13	Zhushali	Medium	32	Angeleno	Late
14	Huangganhe	Medium	33	Wanhuang	Late
15	Jisheng	Medium	34	Liwang	Late
16	Hongxinli	Medium	35	Friar Plum	Late
17	10-27	Medium	36	Richard Early	Late
18	Dahongpao	Medium	37	Great Rosa	Late
19	Hongbayi	Medium	38	Fortune	Late

**Table 2:** Aroma component contents in 38 plum germplasm resources.

**Aroma Components**	**Content (μg/kg)**								
	**Hongshou**	**Lixingzazhong**	**Zaoli**	**Jizaofeng**	**Dadanguo**	**Xiangjiaoli**	**Homgxing**	**Gaida**	**Jihong**
Pentanal, 2,3-dimethyl-	-	-	49.80 ± 6.12	-	-	-	-	-	-
Propanal	-	-	-	36.35 ± 1.48	-	-	-	-	-
Hexanal, 2-ethyl-	152.57 ± 49.01	-	-	-	29.76 ± 3.27	-	-	-	-
Butanal, 2-methyl-	-	-	412.35 ± 32.24	-	-	-	-	-	-
4-Pentenal, 2-ethyl-	8.61 ± 0.71	112.28 ± 3.64	-	-	-	-	-	-	-
2-Pentenal, (E)-	-	-	-	-	-	153.50 ± 56.58	80.15 ± 0.53	108.07 ± 9.35	401.19 ± 28.28
3-Hexenal	-	-	-	-	-	20,369.63 ± 320.81	21,632.75 ± 1267.63	-	-
4-Pentenal, 2-methyl-	1722.80 ± 13.68	1270.84 ± 141.31	6172.56 ± 769.59	1601.62 ± 163.77	-	-	-	-	-
Hexanal	273.84 ± 3.68	382.27 ± 4.36	354.06 ± 16.60	356.06 ± 3.48	349.47 ± 111.57	15,406.95 ± 677.32	-	-	21,599.29 ± 1349.80
2-Hexenal, (E)-	17,973.41 ± 93.52	28,350.34 ± 834.29	14,427.18 ± 1436.51	12,610.73 ± 313.99	30.60 ± 3.63	23.49 ± 1.04	71.12 ± 3.96	7663.33 ± 327.72	541.52 ± 1.62
2-Hexenal	-	-	-	109.91 ± 11.89	4336.98 ± 33.15	8434.42 ± 189.33	4739.48 ± 21.09	2493.27 ± 436.56	38,204.4 ± 1032.83
Heptanal	-	-	-	-	28.94 ± 3.95	-	-	-	37.79 ± 0.31
2,4-Hexadienal, (E,E)-	-	-	99.93 ± 17.38	-	-	-	-	-	-
2-Heptenal, (E)-	-	-	-	107.47 ± 18.96	101.73 ± 3.43	92.15 ± 8.72	-	-	49.36 ± 4.87
2-Heptenal, (Z)-	-	-	328.62 ± 15.98	1000.13 ± 80.89	-	-	-	-	-
Benzaldehyde	-	-	-	-	132.92 ± 19.60	-	824.78 ± 33.81	-	164.74 ± 4.68
4-Oxohex-2-enal	-	-	-	-	-	215.60 ± 8.11	-	-	-
Hexanal dimethyl acetal	-	-	-	-	-	-	-	-	-
2,4-Heptadienal, (E,E)-	-	-	-	-	-	-	-	-	-
Benzeneacetaldehyde	-	-	34.80 ± 8.14	77.70 ± 4.79	-	34.82 ± 1.24	-	-	-
2-Octenal, (E)-	65.27 ± 21.91	-	244.38 ± 55.35	158.18 ± 5.05	28.13 ± 1.18	85.60 ± 3.71	-	-	-
Nonanal	-	-	-	-	83.04 ± 5.09	497.04 ± 3.44	339.57 ± 5.64	229.75 ± 30.78	218.8 ± 3.06
2,5-Dihydroxybenzaldehyde, 2TMS derivative	-	132.54 ± 11.89	-	31.31 ± 0.77	-	95.57 ± 0.90	77.06 ± 0.93	-	-
1-Cyclohexene-1-carboxaldehyde, 2,6,6-trimethyl-	-	-	-	-	-	-	-	-	-
3-Cyclohexene-1-acetaldehyde, .alpha.,4-dimethyl-	-	-	-	-	82.98 ±3.04	-	-	-	-
Octanal	-	-	-	-	-	-	84.05 ± 4.05	-	-
Trimethylene oxide	-	-	-	-	-	35.72 ± 3.24	30.85 ± 0.76	-	-
Acetic acid, methyl ester	-	-	-	-	-	-	-	33.89 ± 5.59	-
Ethyl acetate	-	-	-	-	-	3623.85 ± 74.17	-	-	-
Formic acid, butyl ester	-	-	2964.87 ± 87.90	-	-	1565.48 ± 48.61	-	-	-
n-Propyl acetate	93.49 ± 0.51	89.29 ± 5.11	-	188.55 ± 3.16	444.12 ± 16.23	621.68 ± 21.86	25.08 ± 2.94	-	-
Butanoic acid, methyl ester	-	-	312.95 ± 15.09	-	144.85 ± 11.52	-	-	143.37 ± 11.33	245.49 ± 4.96
Isobutyl acetate	-	-	-	-	243.21 ± 23.24	-	-	-	-
Butanoic acid, ethyl ester	-	-	-	-	-	-	-	8760.45 ± 253.06	-
Propanoic acid, propyl ester	-	-	-	-	38.09 ± 24.67	-	-	-	-
Acetic acid, butyl ester	-	-	14.40 ± 3.04	-	-	34,538.34 ± 1315.61	-	2824.64 ± 393.59	-
Butanoic acid, 2-methyl-, ethyl ester	-	-	-	-	-	19.87 ± 3.66	-	-	-
1-Butanol, 3-methyl-, acetate	-	-	944.45 ± 30.04	-	-		-	-	-
3-Methyl-3-buten-1-ol, acetate	-	-	-	-	947.57 ± 32.28		-	-	-
2-Propenoic acid, butyl ester	-	-	12.51 ± 1.65	-	-	69.61± 5.73	68.33 ± 4.09	-	-
Butanoic acid, propyl ester	-	-	-	-	53.12 ± 0.85	112.13 ± 1.16	-	-	-
Butanoic acid, hexyl ester	-	-	-	-	-	-	-	-	-
Pentanoic acid, ethyl ester	-	-	-	-	-	-	-	11.76 ± 1.96	-
Propanoic acid, butyl ester	-	-	63.78 ± 1.41	-	5005.90 ± 44.20	2898.21 ± 48.13	-	-	-
Acetic acid, pentyl ester	-	-	113.65 ± 18.80	-	6644.36 ± 47.22	1889.52 ± 46.17	-	-	-
2-Buten-1-ol, 3-methyl-, acetate	-	-	-	-	-	-	-	56.64 ± 9.98	-
Hexanoic acid, methyl ester	-	-	-	-	604.52 ± 15.65	124.34 ± 16.78	-	-	-
Propanoic acid, 2-methyl-, butyl ester	-	-	-	-	43.30 ± 2.32	88.87 ± 0.50	-	-	-
1-Butanol, 3-methyl-, propanoate	-	-	-	-	111.62 ± 11.29	-	-	-	-
Butanoic acid, butyl ester	-	-	-	-	14,733.00 ± 685.44	17,662.76 ± 97.42	-	-	-
Hexanoic acid, ethyl ester	-	17,714.55 ± 784.53	15,672.17 ± 142.31	14,564.40 ± 632.52	-	-	143.91 ± 7.48	-	-
4-Hexen-1-ol, acetate	36.56 ± 30.69	5751.02 ± 200.18	16,507.64 ± 1207.29	2676.42 ± 134.38	-	-	-	-	-
3-Hexen-1-ol, acetate, (Z)-	503.89 ± 36.67	2239.93 ± 118.28	3390.08 ± 350.25	100.43 ± 7.49	15,560.94 ± 314.76	15,243.43 ± 119.86	255.47 ± 120.86	10,526.02 ± 1129.03	767.83 ± 95.69
Acetic acid, hexyl ester	708.29 ± 16.24	1014.83 ± 235.97	-	119.49 ± 0.92	97,209.83 ± 1364.12	10,516.06 ± 472.56	196.93 ± 57.6	1617.78 ± 1041.96	63.85 ± 10.80
2-Hexen-1-ol, acetate, (Z)-	-	-	-	-	-	-	-	2965.68 ± 1151.45	-
Butyl 2-methylbutanoate	-	-	-	-	1227.61 ± 61.73	683.54 ± 24.81	-	-	-
Butanoic acid, 3-methyl-, butyl ester	-	-	42.59 ± 0.94	-	-	28.17 ± 2.63	-	-	-
2(3H)-Furanone, 5-ethyldihydro-	-	-	-	-	64.64 ± 5.81	-	-	-	-
Butanoic acid, 2-methylbutyl ester	-	-	-	-	79.75 ± 8.19	78.45 ± 4.41	-	-	-
Butanoic acid, pentyl ester	-	-	-	-	168.23 ± 6.93	-	-	-	-
Pentanoic acid, pentyl ester	-	-	-	-	-	115.54 ± 7.03	-	-	-
Hexanoic acid, propyl ester	-	-	-	-	-	-	-	-	-
Linalyl acetate	-	-	-	-	-	-	-	-	-
1,6-Octadien-3-ol, 3,7-dimethyl-, formate	-	-	-	-	-	-	-	-	-
Propanoic acid, hexyl ester	-	-	-	-	1077.15 ± 11.65	147.77 ± 3.36	-	-	-
Acetic acid, heptyl ester	-	-	-	-	229.34 ± 2.03	-	-	-	-
Octanoic acid, methyl ester	-	-	-	-	-	-	-	-	-
Acetic acid, 2-ethylhexyl ester	-	-	-	-	-	-	-	-	-
Acetic acid, phenylmethyl ester	-	-	-	-	-	-	-	-	-
Butanoic acid, 3-hexenyl ester, (Z)-	-	-	-	-	-	562.74 ± 48.33	-	-	-
Butanoic acid, 3-hexenyl ester, (E)-	-	-	-	-	-	-	-	-	-
Methyl salicylate	-	-	-	-	-	-	-	-	-
Hexanoic acid, butyl ester	-	-	-	-	10,176.27 ± 586.98	1562.76 ± 48.32	-	-	-
Propanoic acid, 2-methyl-, hexyl ester	-	-	-	-	-	-	-	-	-
Octanoic acid, ethyl ester	-	-	-	-	-	-	-	285.27 ± 97.67	-
Acetic acid, octyl ester	-	-	-	-	289.39 ± 49.26	-	-	-	-
1,4-Butanediol, diacetate	-	-	-	-	-	-	-	-	-
cis-3-Hexenyl-.alpha.-methylbutyrate	-	-	-	-	41.78 ± 1.61	-	-	-	-
Butanoic acid, 2-methyl-, hexyl ester	-	-	-	-	137.01 ± 10.01	-	-	-	-
2(3H)-Furanone, 5-butyldihydro-	-	-	-	-	-	-	-	-	-
Hexanoic acid, 2-methylbutyl ester	-	-	-	-	44.53 ± 14.84	-	-	-	-
Hexanoic acid, pentyl ester	-	-	-	-	-	-	-	-	-
Geranyl isovalerate	-	-	-	-	-	-	-	-	-
Ethyl trans-4-decenoate	-	-	-	-	-	-	-	-	-
Hexanoic acid, hexyl ester	-	-	-	-	31.14 ± 3.54	-	-	-	-
Butyl caprylate	-	58.59 ± 8.10	147.26 ± 9.12	-	-	-	-	-	-
2(3H)-Furanone, 5-hexyldihydro-	-	-	50.67 ± 10.18	79.37 ± 45.66	229.62 ± 27.28	18.85 ± 4.27	-	-	-
2,2,4-Trimethyl-1,3-pentanediol diisobutyrate	-	188.50 ± 12.26	208.48 ± 1.16	334.82 ± 13.23	56.90 ± 22.09	-	29.08 ± 7.86	-	-
Benzoic acid, 2-ethylhexyl ester	-	-	-	-	248.07 ± 8.46	57.03 ± 11.05	265.79 ± 14.29	-	-
CH_3_C(O)CH_2_CH_2_OH	-	-	-	-	132.03 ± 14.21	-	-	12,508.42 ± 105.45	4944.7 ± 163.89
Methyl Isobutyl Ketone	-	-	-	-	-	1041.88 ± 18.18	1091.16 ± 34.88	-	-
6-Hepten-3-one, 4-methyl-	-	-	-	-	-	-	-	-	-
1-Hepten-3-one	-	-	19.93 ± 7.15	-	-	-	-	-	-
1-Octen-3-one	36.21 ± 12.31	-	-	-	-	-	-	-	-
3-Heptanone, 5-methyl-	-	-	-	-	-	-	-	-	-
5-Hepten-2-one, 6-methyl-	36.65 ± 0.98	108.10 ± 22.75	-	204.91 ± 11.10	240.39 ± 5.84	-	-	-	-
3-Octanone	-	304.50 ± 22.81	-	-	-	-	-	117.67 ± 27.95	84.02 ± 5.8
Cyclohexanone, 2,2,6-trimethyl-	-	-	-	-	-	-	-	-	-
Isophorone	-	-	-	-	-	-	-	-	-
Ionone	17.64 ± 3.98	-	50.94 ± 1.52	30.45 ± 3.15	-	-	-	-	-
2-Buten-1-one, 1-(2,6,6-trimethyl-1,3-cyclohexadien-1-yl)-, (E)-	-	-	-	-	-	-	-	-	49.29 ± 2.40
1(2H)-Naphthalenone, 3,4-dihydro-3,3,6,8-tetramethyl-	112.31 ± 3.40	405.35 ± 75.55	-	263.43 ± 5.83	16.69 ± 5.30	32.94 ± 8.78	19.01 ± 6.17	-	30.46 ± 1.76
3-Buten-2-ol, 2-methyl-	-	-	-	-	-	-	-	-	-
1-Butanol	-	-	-	-	3149.81 ± 28.52	-	-	-	-
Cyclohexanol, 4-methyl-, trans-	-	-	456.55 ± 37.60	-	-	-	-	-	-
5-Hexen-2-ol, 5-methyl-	-	-	-	-	-	-	-	-	2146.75 ± 131.99
Cyclohexanol, 4-methyl-, cis-	-	-	-	-	-	-	-	-	-
Cyclohexanol, 4-methyl-	-	-	56.7.26	-	-	-	-	-	-
1-Butanol, 2-methyl-	-	-	-	-	2785.84 ± 16.10	-	-	-	164.39 ± 8.78
1-Butanol, 3-methyl-	-	-	-	-	110.53 ± 2.80	-	-	-	-
1-Pentanol	-	-	-	-	-	-	-	-	-
2-Penten-1-ol, (Z)-	-	-	-	-	-	-	-	-	-
3-Hexen-1-ol, (E)-	-	-	-	-	-	-	-	-	-
4-Hexen-1-ol, (E)-	3489.61 ± 34.99	3176.33 ± 27.05	3013.53 ± 124.50	3053.13 ± 29.71	8104.76 ± 27.55	15,333.59 ± 316.98	11,513.62 ± 94.48	-	-
2-Hexen-1-ol, (E)-	-	6718.50 ± 156.05	8573.39 ± 65.54	4946.46 ± 186.00	-	-	-	2061.03 ± 32.19	3258.99 ± 51.56
1-Hexanol	-	-	-	-	-	2232.43 ± 47.09	2486.5 ± 92.96	1566.73 ± 186.81	10,440.43 ± 424.24
1-Butanol, 2-methyl-, acetate	-	-	-	-	1643.65 ± 26.18	-	-	-	-
Ethanol, 2-butoxy-	-	-	-	-	-	35.65 ± 1.16	53.48 ± 2.54	-	-
4-Penten-1-ol, propanoate	-	14.00 ± 8.28	-	-	-	223.11 ± 64.34	-	-	-
3-Hexanol, 3-ethyl-	-	14.37 ± 3.33	30.84 ± 1.23	-	-	-	-	-	-
3-Heptanol	-	36.90 ± 3.92	37.74 ± 7.55	44.46 ± 0.98	-	-	-	-	20.00 ± 3.72
1-Octen-3-ol	527.69 ± 10.51	409.97 ± 5.16	1506.24 ± 56.88	1181.25 ± 33.07	-	-	-	-	29.90 ± 5.27
1-Hexanol, 2-ethyl-	-	-	57.72 ± 11.13	-	828.64 ± 19.16	1020.91 ± 72.55	130.75 ± 3.54	540.76 ± 13.73	664.13 ± 84.75
1-Octanol	72.96 ± 3.04	5628.93 ± 139.18	1990.82 ± 66.23	1498.60 ± 43.64	327.07 ± 2.27	-	-	-	-
Linalool	-	-	-	-	1153.36 ± 7.74	854.16 ± 27.25	144.59 ± 1.83	885.72 ± 58.52	436.63 ± 3.22
1,5,7-Octatrien-3-ol, 3,7-dimethyl-	-	-	-	-	297.68 ± 15.54	-	-	-	184.38 ± 5.90
Hotrienol	-	-	-	-	-	-	-	-	-
trans-Ocimenol	-	-	6396.37 ± 159.03	2753.61 ± 149.34	-	-	-	-	-
L-.alpha.-terpineol	-	4188.96 ± 274.17	-	-	-	-	-	170.47 ± 24.32	273.82 ± 22.26
alpha.-terpineol	-	-	-	-	-	-	-	273.82 ± 22.26	241.67 ± 7.46
3-Buten-1-ol, 3-methyl-	-	60.90 ± 1.53	-	-	-	-	-	-	-
2,6-Octadien-1-ol, 3,7-dimethyl-, (Z)-	-	-	9078.43 ± 286.27	-	-	-	-	-	-
Pentanoic acid, 3-methyl-4-oxo-	-	-	-	-	706.85 ± 29.16	1167.94 ± 24.23	2546.43 ± 41.8	-	-
Butanoic acid	-	-	-	-	153.75 ± 63.23	-	-	-	-
1-Hexyne, 5-methyl-	-	-	-	-	-	-	113.62 ± 16.24	-	-
4-Octenoic acid, ethyl ether	-	-	172.24 ± 21.33	-	-	-	-	34.86 ± 3.83	-
Phenol, 4-(2-propenyl)-	-	-	-	-	-	-	-	-	-
Methyleugenol	-	-	-	-	152.73 ± 6.26	-	-	-	-
**Aroma Components**	**Content (μg/kg)**								
	**Taiyang**	**Taoxingli**	**Aitianli**	**Zhushali**	**Huangganhe**	**Jisheng**	**Hongxinli**	**10-27**	**Dahongpao**
Pentanal, 2,3-dimethyl-	-	-	-	-	-	-	-	-	-
Propanal	-	-	-	-	40.91 ± 1.11	-	-	-	-
Hexanal, 2-ethyl-	-	-	-	-	-	-	-	-	-
Butanal, 2-methyl-	-	-	-	-	-	-	-	-	-
4-Pentenal, 2-ethyl-	-	-	-	-	-	623.90 ± 16.32	-	-	-
2-Pentenal, (E)-	-	308.76 ± 27.29	231.63 ± 7.45	-	-	657.32 ± 10.34	-	-	84.77 ± 1.78
3-Hexenal	-	-	-	-	-	-	-	-	-
4-Pentenal, 2-methyl-	-	-	-	-	-	-	-	-	-
Hexanal	4604.67 ± 359.44	6415.49 ± 698.66	8541.49 ± 325.55	573.23 ± 11.10	1642.63 ± 59.06	1093.37 ± 193.70	140.56 ± 9.75	446.07 ± 21.20	22,491.20 ± 1068.91
2-Hexenal, (E)-	686.84 ± 2.42	612.72 ± 11.24	576.54 ± 19.40	53.85 ± 1.33	301.40 ± 15.54	208.80 ± 11.24	94.69 ± 0.68	92.87 ± 2.84	469.71 ± 22.67
2-Hexenal	40,699.2 ± 273.44	21,022.9 ± 1605.33	42,142.37 ± 1292.95	3137.06 ± 281.47	9341.52 ± 141.89	11,935.11 ± 709.91	3538.8 ± 30.37	9166.63 ± 551.69	13,184.21 ± 347.94
Heptanal	-	42.73 ± 12.92	-	-	18.7 ± 0.55	36.89 ± 3.78	-	-	-
2,4-Hexadienal, (E,E)-	-	-	-	-	23.44 ± 9.26	-	-	-	-
2-Heptenal, (E)-	55.55 ± 3.41	84.84 ± 11.74	-	34.15 ± 23.03	93.23 ± 4.75	36.91 ± 0.89	46.15 ± 1.41	33.05 ± 7.25	21.70 ± 3.95
2-Heptenal, (Z)-	-	-	80.54 ± 6.45	23.89 ± 5.33	97.34 ± 2.90	-	-	-	-
Benzaldehyde	35.77 ± 0.65	377.03 ± 23.02	184.38 ± 56.12	451.97 ± 47.98	890.27 ± 47.27	89.34 ± 3.69	119.04 ± 5.48	-	-
4-Oxohex-2-enal	-	-	-	-	-	-	-	-	-
Hexanal dimethyl acetal	-	-	-	-	-	-	-	-	-
2,4-Heptadienal, (E,E)-	-	-	-	-	1218.00 ± 246.95	1033.88 ± 3.91	-	-	-
Benzeneacetaldehyde	-	-	-	-	-	-	-	-	-
2-Octenal, (E)-	-	137.03 ± 27.46	-	-	63.37 ± 9.65	53.76 ± 28.67	-	-	-
Nonanal	72.44 ± 2.21	51.64 ± 31.06	49.59 ± 8.58	-	5.84 ± 0.17	9.13 ± 1.43	-	-	32.47 ± 1.97
2,5-Dihydroxybenzaldehyde, 2TMS derivative	-	-	-	-	-	-	-	-	-
1-Cyclohexene-1-carboxaldehyde, 2,6,6-trimethyl-	-	-	-	-	757.12 ± 15.76	162.82 ± 120.35	-	-	-
3-Cyclohexene-1-acetaldehyde, .alpha.,4-dimethyl-	-	-	-	-	585.03 ± 72.77	428.12 ± 384.90	132.00 ± 22.39	-	-
Octanal	-	-	-	-	-	-	-	-	-
Trimethylene oxide	-	25.78 ± 2.63	-	27.53 ± 3.09	-	-	-	-	-
Acetic acid, methyl ester	102.15 ± 1.44	36.48 ± 5.73	-	-	-	-	-	-	-
Ethyl acetate	-	-	-	-	-	-	-	-	-
Formic acid, butyl ester	-	-	-	-	-	-	-	-	-
n-Propyl acetate	525.00 ± 11.56	628.39 ± 183.96	-	-	-	-	248.49 ± 3.72	-	-
Butanoic acid, methyl ester	256.65 ± 9.86	254.62 ± 49.69	84.69 ± 7.41	196.34 ± 5.72	175.48 ± 6.22	182.00 ± 3.79	127.04 ± 2.50	76.26 ± 6.37	71.33 ± 3.76
Isobutyl acetate	590.63 ± 47.16	260.61 ± 58.16	-	-	-	-	127.91 ± 6.86	-	-
Butanoic acid, ethyl ester	-	-	-	-	-	-	-	-	-
Propanoic acid, propyl ester	-	-	-	-	-	-	-	-	-
Acetic acid, butyl ester	1667.87 ± 122.46	6674.75 ± 2937.46	-	552.02 ± 77.59	-	-	1550.72 ± 55.86	3088.38 ± 126.02	3750.75 ± 470.72
Butanoic acid, 2-methyl-, ethyl ester	-	-	-	-	-	-	-	-	-
1-Butanol, 3-methyl-, acetate	1149.3 ± 46.32	-	-	-	-	-	427.86 ± 28.18	-	-
3-Methyl-3-buten-1-ol, acetate	392.1 ± 24.42	-	-	-	-	-	425.05 ± 10.05	-	55.86 ± 13.65
2-Propenoic acid, butyl ester	-	-	-	-	-	-	-	-	-
Butanoic acid, propyl ester	-	-	-	-	-	-	-	-	-
Butanoic acid, hexyl ester	-	-	-	-	-	-	-	-	-
Pentanoic acid, ethyl ester	-	-	-	-	-	-	-	-	-
Propanoic acid, butyl ester	-	-	-	-	-	-	-	-	-
Acetic acid, pentyl ester	-	-	-	-	-	-	-	-	-
2-Buten-1-ol, 3-methyl-, acetate	86.82 ± 10.62	-	-	-	-	-	622.27 ± 27.13	-	58.71 ± 9.34
Hexanoic acid, methyl ester	-	-	-	-	-	-	-	-	-
Propanoic acid, 2-methyl-, butyl ester	-	-	-	-	-	-	-	-	-
1-Butanol, 3-methyl-, propanoate	-	-	-	-	-	-	-	-	-
Butanoic acid, butyl ester	-	-	-	-	-	-	-	-	-
Hexanoic acid, ethyl ester	-	-	-	-	-	-	-	-	-
4-Hexen-1-ol, acetate	-	23,901.46 ± 684.01	-	-	-	-	-	-	15,685.19 ± 353.78
3-Hexen-1-ol, acetate, (Z)-	7920.11 ± 197.49	3798.10 ± 1128.42	17.99 ± 4.30	1096.48 ± 314.78	3969.74 ± 29.15	8188.24 ± 587.98	5517.98 ± 112.55	393.24 ± 37.52	1494.42 ± 34.71
Acetic acid, hexyl ester	2185.18 ± 35.37	545.35 ± 160.70	18.30 ± 5.69	527.17 ± 6.33	512.64 ± 12.23	367.74 ± 19.78	652.38 ± 7.77	219.54 ± 24.85	690.06 ± 11.65
2-Hexen-1-ol, acetate, (Z)-	-	-	-	-	-	-	580.12 ± 64.98	-	309.99 ± 33.19
Butyl 2-methylbutanoate	-	-	-	-	-	-	-	-	-
Butanoic acid, 3-methyl-, butyl ester	-	-	-	-	-	-	-	-	-
2(3H)-Furanone, 5-ethyldihydro-	-	-	-	-	-	-	-	-	-
Butanoic acid, 2-methylbutyl ester	-	-	-	-	-	-	-	-	-
Butanoic acid, pentyl ester	-	-	-	-	-	-	-	-	-
Pentanoic acid, pentyl ester	-	-	-	-	-	-	-	-	-
Hexanoic acid, propyl ester	-	-	-	-	-	-	-	-	-
Linalyl acetate	-	-	-	-	1016.18 ± 51.41	-	-	-	-
1,6-Octadien-3-ol, 3,7-dimethyl-, formate	-	-	-	-	-	-	-	-	-
Propanoic acid, hexyl ester	-	-	-	-	-	-	-	-	-
Acetic acid, heptyl ester	-	-	-	-	-	-	-	-	-
Octanoic acid, methyl ester	-	-	-	-	-	-	-	-	-
Acetic acid, 2-ethylhexyl ester	-	-	-	-	-	-	-	-	-
Acetic acid, phenylmethyl ester	-	-	-	-	-	-	76.98 ± 4.07	-	-
Butanoic acid, 3-hexenyl ester, (Z)-	-	81.23 ± 30.64	-	-	-	54.55 ± 3.98	-	-	-
Butanoic acid, 3-hexenyl ester, (E)-	-	-	-	-	-	-	-	-	-
Methyl salicylate	-	-	-	-	-	-	-	-	-
Hexanoic acid, butyl ester	-	-	-	-	-	-	-	-	-
Propanoic acid, 2-methyl-, hexyl ester	-	-	-	-	-	-	-	-	-
Octanoic acid, ethyl ester	-	-	-	-	-	-	-	-	-
Acetic acid, octyl ester	-	-	-	-	-	-	-	-	-
1,4-Butanediol, diacetate	-	-	-	-	-	-	-	-	-
cis-3-Hexenyl-.alpha.-methylbutyrate	-	-	-	-	-	-	-	-	-
Butanoic acid, 2-methyl-, hexyl ester	-	-	-	-	-	-	-	-	-
2(3H)-Furanone, 5-butyldihydro-	-	-	-	-	-	-	-	-	-
Hexanoic acid, 2-methylbutyl ester	-	-	-	-	-	-	-	-	-
Hexanoic acid, pentyl ester	-	-	-	-	-	-	-	-	-
Geranyl isovalerate	-	-	-	-	-	-	-	-	-
Ethyl trans-4-decenoate	-	-	-	-	-	-	-	-	-
Hexanoic acid, hexyl ester	-	-	-	-	-	-	-	-	-
Butyl caprylate	-	-	-	-	-	-	-	-	-
2(3H)-Furanone, 5-hexyldihydro-	-	-	-	-	-	-	-	-	-
2,2,4-Trimethyl-1,3-pentanediol diisobutyrate	51.12 ± 8.53	-	-	-	-	-	-	-	-
Benzoic acid, 2-ethylhexyl ester	543.64 ± 12.01	423.79 ± 19.07	439.13 ± 5.18	-	41.79 ± 8.14	76.57 ± 5.05	125.67 ± 5.39	122.48 ± 1.93	95.25 ± 12.62
CH_3_C(O)CH_2_CH_2_OH	20,948.8 ± 261.23	-	-	-	-	-	-	-	-
Methyl Isobutyl Ketone	-	-	-	-	-	-	-	-	-
6-Hepten-3-one, 4-methyl-	-	-	-	-	-	384.49 ± 7.99	-	-	-
1-Hepten-3-one	-	-	-	-	-	-	1170.37 ± 31.01	-	-
1-Octen-3-one	-	-	-	-	-	-	-	-	-
3-Heptanone, 5-methyl-	-	-	-	-	-	-	-	-	-
5-Hepten-2-one, 6-methyl-	-	-	-	-	-	163.37 ± 4.81	94.17 ± 6.19	-	-
3-Octanone	79.25 ± 2.41	159.54 ± 17.15	-	-	-	-	-	-	66.47 ± 7.11
Cyclohexanone, 2,2,6-trimethyl-	-	-	-	-	-	-	-	-	-
Isophorone	-	-	-	-	-	21.90 ± 13.10	-	-	-
Ionone	-	-	-	-	-	55.12 ± 4.96	-	-	-
2-Buten-1-one, 1-(2,6,6-trimethyl-1,3-cyclohexadien-1-yl)-, (E)-	174.45 ± 2.64	86.6 ± 3.00	58.05 ± 5.75	-	25.94 ± 5.17	73.96 ± 6.82	-	19.30 ± 2.59	29.42 ± 0.83
1(2H)-Naphthalenone, 3,4-dihydro-3,3,6,8-tetramethyl-	24.17 ± 0.88	22.75 ± 7.24	31.61 ± 8.18	-	-	-	-	-	-
3-Buten-2-ol, 2-methyl-	-	-	-	-	-	-	-	-	-
1-Butanol	-	-	-	-	-	-	-	-	-
Cyclohexanol, 4-methyl-, trans-	-	-	-	-	-	-	-	-	-
5-Hexen-2-ol, 5-methyl-	-	-	1040.35 ± 52.07	-	-	-	-	418.81 ± 36.60	-
Cyclohexanol, 4-methyl-, cis-	-	-	-	-	-	-	-	-	-
Cyclohexanol, 4-methyl-	-	-	-	-	-	-	-	-	369.62 ± 5.88
1-Butanol, 2-methyl-	96.07 ± 2.35	-	-	-	-	-	-	73.70 ± 20.63	-
1-Butanol, 3-methyl-	-	-	-	-	-	-	-	-	-
1-Pentanol	-	-	189.51 ± 8.55	213.46 ± 12.15	-	-	199.96 ± 44.63	107.59 ± 49.41	-
2-Penten-1-ol, (Z)-	-	-	-	-	-	-	-	-	-
3-Hexen-1-ol, (E)-	-	-	-	8308.08 ± 450.14	-	-	-	-	-
4-Hexen-1-ol, (E)-	-	-	-	-	21,349.51 ± 322.98	20,099.05 ± 757.18	8000.09 ± 314.36	-	-
2-Hexen-1-ol, (E)-	4506.23 ± 50.62	4346.67 ± 80.23	-	1632.94 ± 23.10	2953.75 ± 60.04	3767.29 ± 153.10	1461.57 ± 41.72	1532.51 ± 14.00	3706.00 ± 139.72
1-Hexanol	5385.17 ± 92.50	6530.28 ± 216.47	4945.03 ± 173.89	6295.61 ± 184.64	6892.34 ± 157.41	5613.09 ± 270.39	2209.26 ± 68.84	6217.01 ± 43.23	8806.42 ± 57.90
1-Butanol, 2-methyl-, acetate	-	-	-	-	-	-	-	-	-
Ethanol, 2-butoxy-	-	-	-	-	-	-	-	-	-
4-Penten-1-ol, propanoate	-	-	-	-	-	-	-	-	-
3-Hexanol, 3-ethyl-	-	33.46 ± 1.33	-	-	-	-	-	-	-
3-Heptanol	32.26 ± 1.32	-	57.06 ± 4.99	-	27.5.25	13.21 ± 2.95	-	-	20.19 ± 0.59
1-Octen-3-ol	31.75 ± 2.26	26.83 ± 7.3	-	-	-	34.89 ± 2.39	25.47 ± 5.75	26.54 ± 8.26	-
1-Hexanol, 2-ethyl-	795.83 ± 6.07	2481.78 ± 217.49	1641.14 ± 28.26	721.84 ± 315.16	749.90 ± 38.38	1527.99 ± 38.00	812.29 ± 2.82	600.27 ± 14.89	1579.89 ± 20.48
1-Octanol	-	-	15.74 ± 10.91	-	-	-	128.51 ± 3.88	-	-
Linalool	126.40 ± 15.61	3356.43 ± 362.25	303.55 ± 13.50	-	995.82 ± 89.09	1312.88 ± 69.64	107.32 ± 4.92	114.30 ± 3.50	114.08 ± 7.25
1,5,7-Octatrien-3-ol, 3,7-dimethyl-	245.19 ± 4.05	-	-	-	-	-	-	58.31 ± 7.90	-
Hotrienol	-	-	-	-	-	-	-	-	-
trans-Ocimenol	-	-	-	-	-	30.64 ± 19.44	-	-	-
L-.alpha.-terpineol	-	-	646.77 ± 37.68	-	-	152.22 ± 9.85	305.33 ± 41.23	-	-
alpha.-terpineol	109.33 ± 16.97	-	-	-	159.74 ± 4.18	-	-	-	-
3-Buten-1-ol, 3-methyl-	-	-		144.78 ± 16.00	-	-	-	-	10.28 ± 4.64
2,6-Octadien-1-ol, 3,7-dimethyl-, (Z)-	-	35.18 ± 18.63	-	-	-	-	-	-	-
Pentanoic acid, 3-methyl-4-oxo-	-	-	-	-	-	-	-	-	-
Butanoic acid	-	-	-	-	-	-	-	-	-
1-Hexyne, 5-methyl-	-	-	-	-	-	-	-	-	-
4-Octenoic acid, ethyl ether	-	-	-	-	-	-	-	-	-
Phenol, 4-(2-propenyl)-	-	-	-	-	-	-	-	-	-
Methyleugenol	-	-	28.01 ± 3.89	-	-	-	-	-	-
**Aroma Components**	**Content (μg/kg)**								
	**Hongbayi**	**Jinxixiangjiaoli**	**Heibali**	**Suili 3**	**Qiuji**	**98-1-6**	**XY**	**XY7-2**	**XY7-1**
Pentanal, 2,3-dimethyl-	-	-	-	-	-	-	-	-	-
Propanal	-	-	-	-	-	-	-	-	-
Hexanal, 2-ethyl-	-	-	-	-	-	-	-	-	-
Butanal, 2-methyl-	-	-	-	-	-	-	-	-	-
4-Pentenal, 2-ethyl-	-	-	-	-	-	-	-	-	-
2-Pentenal, (E)-				121.72 ± 1.19	119.57 ± 3.88	16.74 ± 9.16	28.95 ± 4.37	36.54 ± 3.38	65.54 ± 3.93
3-Hexenal	-	-	-	-	-	-	-	-	-
4-Pentenal, 2-methyl-	-	-	1480.04 ± 29.65	-	-	1453.79 ± 88.68	-	-	-
Hexanal	388.03 ± 26.57	-	-	-	12,491.9 ± 811.98	-	10,608.98 ± 427.15	8011.46 ± 895.32	4449.98 ± 278.67
2-Hexenal, (E)-	53.05 ± 4.91	17.41 ± 6.62	17.14 ± 1.96	-	1094.78 ± 3.97	159.78 ± 1.09	283.76 ± 2.64	198.07 ± 2.13	86.74 ± 1.60
2-Hexenal	2002.17 ± 82.85	1936.54 ± 37.33	2897.06 ± 43.67	7443.02 ± 70.47	58,329.59 ± 385.00	3797.55 ± 181.83	19,508.33 ± 298.84	5139.65 ± 166.00	2535.56 ± 51.35
Heptanal	-	-	-	33.86 ± 4.59	22.37 ± 2.31	-	-	-	-
2,4-Hexadienal, (E,E)-	-	-	-	-	-	-	-	-	-
2-Heptenal, (E)-	-	57.64 ± 7.76	-	51.96 ± 2.29	-	-	27.41 ± 3.05	-	28.44 ± 3.83
2-Heptenal, (Z)-	-	-	-		126.76 ± 3.66	-	-	-	-
Benzaldehyde	-	24.36 ± 2.04	-	49.48 ± 2.88	-	-	82.57 ± 23.1	53.22 ± 11.34	118.61 ± 2.93
4-Oxohex-2-enal	-	-	-	-	41.19 ± 8.19	-	-	-	-
Hexanal dimethyl acetal	-	-	-	-	-	-	-	-	-
2,4-Heptadienal, (E,E)-	-	-	-	-	-	-	-	-	-
Benzeneacetaldehyde	-	-	-	-	-	-	-	-	-
2-Octenal, (E)-	-	30.78 ± 4.65	-	19.38 ± 0.79	48.68 ± 1.16	-	-	-	-
Nonanal	22.03 ± 13.13	159.62 ± 3.30	-	167.07 ± 5.67	817.40 ± 12.47	39.17 ± 4.49	23.97 ± 5.94	22.94 ± 10.49	-
2,5-Dihydroxybenzaldehyde, 2TMS derivative	-	-	-	-	-	-	-	-	-
1-Cyclohexene-1-carboxaldehyde, 2,6,6-trimethyl-	-	-	117.39 ± 74.48	-	-	-	163.91 ± 123.97	-	172.65 ± 93.80
3-Cyclohexene-1-acetaldehyde, .alpha.,4-dimethyl-	-	-	-	-	-	-	-	-	-
Octanal	-	-	-	-	-	-	-	-	-
Trimethylene oxide	-	-	-	18.32 ± 3.80	-	-	-	-	-
Acetic acid, methyl ester	-	-	-	-	-	-	-	-	-
Ethyl acetate	-	-	-	-	-	-	-	-	-
Formic acid, butyl ester	-	-	-	-	-	-	-	-	-
n-Propyl acetate	97.06 ± 10.13	457.52 ± 3.58	199.33 ± 14.61	3538.78 ± 139.21	67.78 ± 6.46	-	-	-	-
Butanoic acid, methyl ester	69.05 ± 1.86	-	124.98 ± 2.65	-	80.84 ± 0.68	89.46 ± 1.97	79.26 ± 9.94	66.76 ± 1.41	59.07 ± 6.81
Isobutyl acetate	260.61 ± 38.54	677.95 ± 63.09	343.87 ± 48.93	436.85 ± 7.88	-	-	-	-	-
Butanoic acid, ethyl ester	-	-	-	7066.33 ± 512.09	-	-	-	-	-
Propanoic acid, propyl ester	-	-	-	-	-	-	-	-	-
Acetic acid, butyl ester	4188.07 ± 578.37	164,950.91 ± 1013.95	63,154.42 ± 1253.20	16,539.55 ± 300.39	2323.75 ± 203.98	-	-	-	-
Butanoic acid, 2-methyl-, ethyl ester	-	-	-	7.64 ± 3.33	-	-	-	-	-
1-Butanol, 3-methyl-, acetate	-	174.69 ± 5.63	336.07 ± 9.32	817.83 ± 5.12	-	-	-	-	-
3-Methyl-3-buten-1-ol, acetate	510.08 ± 52.78	746.51 ± 21.70	-	443.37 ± 24.63	104.38 ± 9.17	-	-	-	-
2-Propenoic acid, butyl ester	-	-	-	-	-	-	-	-	-
Butanoic acid, propyl ester	-	-	-	99.88 ± 4.41	-	-	-	-	-
Butanoic acid, hexyl ester	-	-	-	-	-	-	-	-	-
Pentanoic acid, ethyl ester	-	-	-	-	-	-	-	-	-
Propanoic acid, butyl ester	-	573.93 ± 2.47	447.02 ± 15.41	-	-	-	-	-	-
Acetic acid, pentyl ester	153.22 ± 21.72	3983.77 ± 116.19	1694.49 ± 34.52	73.03 ± 0.93	39.37 ± 8.42	-	-	-	-
2-Buten-1-ol, 3-methyl-, acetate	120.32 ± 39.52	1485.08 ± 29.86	382.64 ± 57.73	153.88 ± 9.45	181.88 ± 9.35	-	-	-	-
Hexanoic acid, methyl ester	-	-	80.92 ± 5.92	-	-	-	-	-	-
Propanoic acid, 2-methyl-, butyl ester	-	27.8 ± 2.21	33.94 ± 2.54	-	-	-	-	-	-
1-Butanol, 3-methyl-, propanoate	-	-	-	-	-	-	-	-	-
Butanoic acid, butyl ester	-	-	-	-	-	-	-	-	-
Hexanoic acid, ethyl ester	-	-	-	4036.88 ± 306.38	-	-	-	-	-
4-Hexen-1-ol, acetate	-	22.75 ± 6.71	-	15,378.75 ± 118.73	-	-	-	7844.29 ± 106.40	-
3-Hexen-1-ol, acetate, (Z)-	562.18 ± 6.54	7385.23 ± 182.37	2475.48 ± 36.75	4306.67 ± 114.70	414.43 ± 19.47	54.38 ± 7.52	-	-	-
Acetic acid, hexyl ester	1727.57 ± 34.27	137,368.35 ± 5459.64	49,451.21 ± 796.81	3365.31 ± 124.09	447.06 ± 2.29	30.18 ± 21.46	-	-	-
2-Hexen-1-ol, acetate, (Z)-	-	-	-	-	277.47 ± 1.23	-	-	-	-
Butyl 2-methylbutanoate	-	29.36 ± 1.17	228.69 ± 13.46	-	-	-	-	-	-
Butanoic acid, 3-methyl-, butyl ester	-	-	-	-	-	-	-	-	-
2(3H)-Furanone, 5-ethyldihydro-	-	-	-	-	-	-	-	-	-
Butanoic acid, 2-methylbutyl ester	-	-	-	-	-	-	-	-	-
Butanoic acid, pentyl ester	-	-	175.39 ± 8.30	-	-	-	-	-	-
Pentanoic acid, pentyl ester	-	-	-	-	-	-	-	-	-
Hexanoic acid, propyl ester	-	-	-	32.15 ± 4.12	-	-	-	-	-
Linalyl acetate	-	-	-	-	-	-	-	-	-
1,6-Octadien-3-ol, 3,7-dimethyl-, formate	-	-	-	-	-	-	-	-	-
Propanoic acid, hexyl ester	-	91.38 ± 2.03	97.74 ± 7.57	-	-	-	-	-	-
Acetic acid, heptyl ester	29.67 ± 6.61	220.57 ± 6.00	71.89 ± 2.35	42.52 ± 5.17	-	-	-	-	-
Octanoic acid, methyl ester	-	36.92 ± 16.12	-	-	-	-	-	-	-
Acetic acid, 2-ethylhexyl ester	-	-	-	-	64.2 ± 6.94	34.66 ± 9.00	-	-	-
Acetic acid, phenylmethyl ester	-	66.44 ± 11.34	-	-	-	-	-	-	-
Butanoic acid, 3-hexenyl ester, (Z)-	-	-	-	49.59 ± 5.75	55.15 ± 19.02	-	-	-	-
Butanoic acid, 3-hexenyl ester, (E)-	-	-	78.61 ± 0.96	35.98 ± 19.41	-	-	-	-	-
Methyl salicylate	-	-	-	-	-	-	-	-	-
Hexanoic acid, butyl ester	-	2318.62 ± 45.27	5936.98 ± 234.16	421.31 ± 5.70	-	-	-	-	-
Propanoic acid, 2-methyl-, hexyl ester	-	-	-	-	-	-	-	-	-
Octanoic acid, ethyl ester	-	-	-	186.70 ± 12.26	-	-	-	-	-
Acetic acid, octyl ester	288.14 ± 33.05	1163.14 ± 51.48	283.05 ± 13.54	301.97 ± 24.98	-	-	-	-	-
1,4-Butanediol, diacetate	-	152.55 ± 102.01	-	-	-	-	-	-	-
cis-3-Hexenyl-.alpha.-methylbutyrate	-	-	-	-	-	-	-	-	-
Butanoic acid, 2-methyl-, hexyl ester	-	-	123.22 ± 1.89	-	-	-	-	-	-
2(3H)-Furanone, 5-butyldihydro-	-	27.47 ± 2.82	-	-	-	-	-	-	-
Hexanoic acid, 2-methylbutyl ester	-	-	45.40 ± 8.63	-	-	-	-	-	-
Hexanoic acid, pentyl ester	-	-	32.27 ± 2.36	-	-	-	-	-	-
Geranyl isovalerate	-	62.82 ± 6.72	-	-	-	-	-	-	-
Ethyl trans-4-decenoate	-	-	-	-	-	-	-	-	-
Hexanoic acid, hexyl ester	-	78.37 ± 2.18	230.02 ± 27.71	-	-	-	-	-	-
Butyl caprylate	-	193.19 ± 2.49	26.02 ± 1.34	-	-	-	-	-	-
2(3H)-Furanone, 5-hexyldihydro-	48.90 ± 8.86	40.18 ± 1.45	-	111.22 ± 8.82	53.92 ± 2.05	-	-	-	-
2,2,4-Trimethyl-1,3-pentanediol diisobutyrate	25.39 ± 3.46	-	-	-	-	-	-	-	-
Benzoic acid, 2-ethylhexyl ester	124.13 ± 10.79	121.36 ± 10.02	139.77 ± 1.91	143.22 ± 0.93	15.69 ± 1.64	167.59 ± 8.59	66.5 ± 6.64	155.45 ± 2.71	160.05 ± 6.05
CH_3_C(O)CH_2_CH_2_OH	15,659.10 ± 137.56	1030.92 ± 14.71	-	11,762.96 ± 262.45	-	-	-	-	-
Methyl Isobutyl Ketone	-	-	-	-	-	-	-	-	-
6-Hepten-3-one, 4-methyl-	-	-	-	-	-	-	-	-	-
1-Hepten-3-one	-	-	-	-	-	-	-	-	-
1-Octen-3-one	-	-	-	-	-	-	-	-	-
3-Heptanone, 5-methyl-	-	-	-	-	-	-	-	-	-
5-Hepten-2-one, 6-methyl-	-	-	104.42 ± 3.23	148.76 ± 25.88	-	-	-	-	-
3-Octanone	42.76 ± 4.24	68.52 ± 2.01	-	-	229.50 ± 1.96	74.56 ± 1.15	123.73 ± 6.23	78.65 ± 1.72	60.87 ± 0.97
Cyclohexanone, 2,2,6-trimethyl-	-	-	-	-	-	-	-	-	-
Isophorone	-	-	-	-	-	-	-	-	-
Ionone	-	-	-	-	-	-	-	-	-
2-Buten-1-one, 1-(2,6,6-trimethyl-1,3-cyclohexadien-1-yl)-, (E)-	-	-	-	26.34 ± 3.91	54.90 ± 1.84	52.65 ± 1.47	-	116.39 ± 4.47	101.77 ± 0.89
1(2H)-Naphthalenone, 3,4-dihydro-3,3,6,8-tetramethyl-	-	-	-	-	-	-	-	-	-
3-Buten-2-ol, 2-methyl-	-	-	-	-	-	-	-	-	-
1-Butanol	-	581.48 ± 22.49	5481.43 ± 62.89	-	-	-	-	-	-
Cyclohexanol, 4-methyl-, trans-	-	-	-	-	-	-	-	186.90 ± 15.10	-
5-Hexen-2-ol, 5-methyl-	-	-	-	-	-	-	-	-	-
Cyclohexanol, 4-methyl-, cis-	-	-	-	-	-	-	-	-	-
Cyclohexanol, 4-methyl-	132.83 ± 6.14	-	-	-	-	278.51 ± 8.04	-	-	310.05 ± 9.13
1-Butanol, 2-methyl-	60.72 ± 8.32	1324.39 ± 68.64	4591.20 ± 80.83	85.14 ± 9.54	-	-	-	-	-
1-Butanol, 3-methyl-	-	-	-	-	-	-	-	-	-
1-Pentanol	-	-	-	-	-	-	-	-	-
2-Penten-1-ol, (Z)-	-	-	-	-	-	-	-	-	-
3-Hexen-1-ol, (E)-	-	-	-	-	-	-	-	-	-
4-Hexen-1-ol, (E)-	5682.33 ± 92.58	1826.78 ± 55.56	6506.82 ± 5.06	-	-	8611.63 ± 309.50	-	-	7591.17 ± 239.57
2-Hexen-1-ol, (E)-	1472.25 ± 30.56		1127.52 ± 5.68	-	6265.47 ± 150.08	2051.77 ± 222.14	2969.41 ± 8.62	2348.98 ± 20.63	1635.39 ± 44.43
1-Hexanol	3623.44 ± 59.04	1979.68 ± 13.37	11,365.38 ± 144.91	3970.07 ± 14.80	10,853.86 ± 54.83	2719.18 ± 254.95	11,536.18 ± 392.71	11,861.90 ± 310.65	12,453.01 ± 414.92
1-Butanol, 2-methyl-, acetate	-	1163.59 ± 68.93	2105.19 ± 226.26	-	-	-	-	-	-
Ethanol, 2-butoxy-	-	-	-	-	-	-	-	-	-
4-Penten-1-ol, propanoate	-	-	-	-	-	-	-	-	-
3-Hexanol, 3-ethyl-	-	-	-	-	-	-	-	-	-
3-Heptanol	-	-	-	15.31 ± 1.16	53.60 ± 9.25	-	-	-	-
1-Octen-3-ol	-	37.45 ± 5.72	-	29.20 ± 1.59	124.81 ± 0.49	-	-	-	-
1-Hexanol, 2-ethyl-	1231.94 ± 36.11	1188.16 ± 29.80	1294.43 ± 5.04	1152.32 ± 48.99	6177.46 ± 237.36	3945.26 ± 8.55	760.80 ± 47.88	1057.86 ± 20.78	853.82 ± 22.81
1-Octanol	-	58.63 ± 6.73	55.88 ± 3.31	26.33 ± 5.04	39.08 ± 2.90	-	-	-	-
Linalool	181.14 ± 1.69	61.57 ± 0.83	207.98 ± 14.03	712.81 ± 41.76	684.61 ± 10.90	279.72 ± 27.17	-	70.23 ± 0.89	-
1,5,7-Octatrien-3-ol, 3,7-dimethyl-	-	34.96 ± 8.68	-	-	-	-	-	-	-
Hotrienol	-	-	-	-	-	-	-	-	-
trans-Ocimenol	-	-	-	-	-	-	-	-	-
L-.alpha.-terpineol	-	-	-	-	-	-	-	-	-
alpha.-terpineol	-	-	-	-	-	-	-	-	-
3-Buten-1-ol, 3-methyl-	-	-	55.26 ± 4.12	-	25.80 ± 1.43	-	-	-	-
2,6-Octadien-1-ol, 3,7-dimethyl-, (Z)-	-	-	-	-	-	-	-	-	-
Pentanoic acid, 3-methyl-4-oxo-	-	301.10 ± 8.52	-	-	-	-	-	-	-
Butanoic acid	-	-	-	-	-	-	-	-	-
1-Hexyne, 5-methyl-	-	-	-	-	-	-	-	-	-
4-Octenoic acid, ethyl ether	-	-	-	-	-	-	-	-	-
Phenol, 4-(2-propenyl)-	-	-	-	-	-	-	-	-	-
Methyleugenol	-	-	-	-	-	-	-	-	-
**Aroma Components**	**Content (μg/kg)**								
	**Xiangguang 2**	**Wanhuang**	**Liwang**	**Friar Plum**	**Richard Early**	**Great Rosa**	**Fortune**		
Pentanal, 2,3-dimethyl-	-	-	-	-	-	-	-		
Propanal	-	-	-	-	-	-	-		
Hexanal, 2-ethyl-	-	-	-	-	-	-	-		
Butanal, 2-methyl-	-	-	-	-	-	-	-		
4-Pentenal, 2-ethyl-	-	-	-	-	-	-	-		
2-Pentenal, (E)-	218.69 ± 53.97	364.05 ± 12.10	-	302.44 ± 3.51	282.89 ± 5.81	259.57 ± 5.58	-		
3-Hexenal	-	-	-	-	-	-	-		
4-Pentenal, 2-methyl-	-	-	-	23,682.84 ± 1171.36	-	-	-		
Hexanal	-	2719.81 ± 109.70	-	-	8327.56 ± 819.13	38,420.80 ± 454.72	-		
2-Hexenal, (E)-	-	543.94 ± 9.40	251.95 ± 2.51	1576.48 ± 22.12	612.41 ± 34.89	2032.69 ± 38.47	-		
2-Hexenal	7310.55 ± 12.74	18,926.68 ± 662.35	7522.10 ± 109.40	42,670.92 ± 207.93	41,164.78 ± 3666.58	136,265.03 ± 1316.20	-		
Heptanal	-	-	-	98.90 ± 1.28	44.63 ± 7.66	99.83 ± 3.63	12.6.63		
2,4-Hexadienal, (E,E)-	-	-	-	99.39 ± 6.23	112.21 ± 9.63	59.21 ± 6.60	880.89 ± 152.20		
2-Heptenal, (E)-	29.87 ± 6.01	-	-	168.01 ± 16.15	65.73 ± 14.98	238.07 ± 14.11	-		
2-Heptenal, (Z)-	-	62.31 ± 1.92	-	-	67.60 ± 5.55	-	-		
Benzaldehyde	-	226.88 ± 16.53	-	84.51 ± 3.80	216.03 ± 7.61	443.48 ± 26.39	-		
4-Oxohex-2-enal	-	-	-	-	-	-	-		
Hexanal dimethyl acetal	-	-	-	-	-	93.69 ± 27.32	-		
2,4-Heptadienal, (E,E)-	-	-	-	-	26.34 ± 5.01	-	-		
Benzeneacetaldehyde	-	-	-	-	37.47 ± 4.19	-	-		
2-Octenal, (E)-	-	-	-	103.45 ± 7.77	54.29 ± 2.82	75.88 ± 7.24	-		
Nonanal	128.20 ± 4.21	35.18 ± 1.04	48.59 ± 9.31	705.33 ± 7.17	760.87 ± 33.92	867.83 ± 61.10	229.76 ± 10.95		
1-Cyclohexene-1-carboxaldehyde, 2,6,6-trimethyl-	-	185.84 ± 37.14	-	-	-	-	-		
3-Cyclohexene-1-acetaldehyde, .alpha.,4-dimethyl-	-	-	-	2642.34 ± 28.62	-	-	-		
Octanal	-	-	-	-	-	-	-		
Trimethylene oxide	-	34.37 ± 2.63	-	19.35 ± 1.44	40.63 ± 4.80	-	-		
Acetic acid, methyl ester	-	-	-	-	-	-	-		
Ethyl acetate	-	-	-	-	-	-	-		
Formic acid, butyl ester	-	-	-	-	-	-	-		
n-Propyl acetate	1519.74 ± 25.89	278.94 ± 17.95	143.64 ± 6.01	-	-	-	177.69 ± 52.50		
Butanoic acid, methyl ester	432.87 ± 25.42	129.82 ± 11.42	86.88 ± 2.44	144.17 ± 4.54	167.23 ± 81.17	145.91 ± 2.13	-		
Isobutyl acetate	160.17 ± 10.44	-	437.64 ± 24.46	-	-	-	169.97 ± 59.70		
Butanoic acid, ethyl ester	5021.79 ± 625.11	-	-	-	-	-	-		
Propanoic acid, propyl ester	-	-	-	-	-	-	-		
Acetic acid, butyl ester	5717.07 ± 203.69	1327.00 ± 47.69	659.32 ± 2.65	-	-	-	4887.11 ± 188.81		
Butanoic acid, 2-methyl-, ethyl ester	-	-	-	-	-	-	-		
1-Butanol, 3-methyl-, acetate	-	-	-	-	-	-	-		
3-Methyl-3-buten-1-ol, acetate	59.36 ± 17.56	23.75 ± 5.77	-	-	-	-	-		
2-Propenoic acid, butyl ester	-	-	-	-	-	-	-		
Butanoic acid, propyl ester	40.26 ± 1.37	-	-	-	-	-	-		
Butanoic acid, hexyl ester		242.96 ± 25.91	-	-	-	-	-		
Pentanoic acid, ethyl ester	-	-	-	-	-	-	-		
Propanoic acid, butyl ester	-	-	-	-	-	-	-		
Acetic acid, pentyl ester	23.55 ± 3.51	-	-	-	-	-	45.47 ± 1.65		
2-Buten-1-ol, 3-methyl-, acetate	34.13 ± 2.40	58.08 ± 2.93	-	-	-	-	37.12 ± 5.03		
Hexanoic acid, methyl ester	-	-	-	-	-	-	-		
Propanoic acid, 2-methyl-, butyl ester	-	-	-	-	-	-	-		
1-Butanol, 3-methyl-, propanoate	-	-	-	-	-	-	-		
Butanoic acid, butyl ester	-	-	-	-	-	-	-		
Hexanoic acid, ethyl ester	3049.16 ± 100.76	-	-	-	-	-	-		
4-Hexen-1-ol, acetate	13,937.56 ± 601.92	25,645.93 ± 132.23	-	45,286.09 ± 1377.07	-	-	-		
3-Hexen-1-ol, acetate, (Z)-	2706.33 ± 76.63	3867.33 ± 405.19	4083.49 ± 91.69	53,023.79 ± 11,112.41	-	-	3732.28 ± 8.77		
Acetic acid, hexyl ester	993.49 ± 4.40	389.53 ± 117.65	88.85 ± 1.72	187.04 ± 22.80	69.54 ± 3.38	72.66 ± 4.96	3056.27 ± 11.02		
2-Hexen-1-ol, acetate, (Z)-	-	-	201.95 ± 5.42	140.66 ± 39.57	-	-	-		
Butyl 2-methylbutanoate	-	-	-	-	-	-	-		
Butanoic acid, 3-methyl-, butyl ester	-	-	-	-	-	-	-		
2(3H)-Furanone, 5-ethyldihydro-	-	-	-	-	-	-	-		
Butanoic acid, 2-methylbutyl ester	-	-	-	-	-	-	-		
Butanoic acid, pentyl ester	-	-	-	-	-	-	-		
Pentanoic acid, pentyl ester	-	-	-	-	-	-	-		
Hexanoic acid, propyl ester	-	-	-	-	-	-	-		
Linalyl acetate	-	-	-	-	-	-	-		
1,6-Octadien-3-ol, 3,7-dimethyl-, formate	-	-	-	-	128.23 ± 30.09	-	-		
Propanoic acid, hexyl ester	-	-	-	-	-	-	-		
Acetic acid, heptyl ester	-	-	-	-	-	-	-		
Octanoic acid, methyl ester	-	-	-	-	-	-	-		
Acetic acid, 2-ethylhexyl ester	-	-	-	87.42 ± 2.29	68.60 ± 8.67	36.52 ± 1.07	38.41 ± 5.91		
Acetic acid, phenylmethyl ester	-	-	-	-	-	-	-		
Butanoic acid, 3-hexenyl ester, (Z)-	-	-	-	82.92 ± 47.41	111.07 ± 9.26	-	-		
Butanoic acid, 3-hexenyl ester, (E)-	-	-	-	-	-	-	-		
Methyl salicylate	-	-	-	-	-	-	-		
Hexanoic acid, butyl ester	109.32 ± 2.07	-	-	-	-	-	386.25 ± 22.94		
Propanoic acid, 2-methyl-, hexyl ester	-	-	-	-	111.60 ± 14.44				
Octanoic acid, ethyl ester	88.88 ± 10.78	-	-	-	-	337.56 ± 122.65	534.05 ± 14.98		
Acetic acid, octyl ester	-	-	-	-	-	-	-		
1,4-Butanediol, diacetate	-	-	-	-	-	-	-		
cis-3-Hexenyl-.alpha.-methylbutyrate	-	-	-	-	-	-	-		
Butanoic acid, 2-methyl-, hexyl ester	-	-	-	-	-	-	-		
2(3H)-Furanone, 5-butyldihydro-	-	-	-	-	-	-	-		
Hexanoic acid, 2-methylbutyl ester	-	-	-	-	-	-	-		
Hexanoic acid, pentyl ester	-	-	-	-	-	-	-		
Geranyl isovalerate	-	-	-	-	-	-	-		
Ethyl trans-4-decenoate	-	-	-	-	-	-	-		
Hexanoic acid, hexyl ester	-	-	-	-	-	-	-		
Butyl caprylate	-	-	-	-	-	-	-		
2(3H)-Furanone, 5-hexyldihydro-	47.34 ± 0.21	-	-	-	-	-	46.07 ± 3.45		
2,2,4-Trimethyl-1,3-pentanediol diisobutyrate	-	-	-	-	-	-	-		
Benzoic acid, 2-ethylhexyl ester	153.78 ± 4.56	151.17 ± 5.27	139.96 ± 9.83	-	187.95 ± 28.09	288.21 ± 6.92	249.55 ± 14.29		
CH_3_C(O)CH_2_CH_2_OH	5791.01 ± 38.32	-	-	-	-	-	-		
Methyl Isobutyl Ketone	-	-	-	-	-	-	-		
6-Hepten-3-one, 4-methyl-	-	-	-	-	-	-	-		
1-Hepten-3-one	-	-	-	-	-	-	-		
1-Octen-3-one	-	-	-	-	-	-	-		
3-Heptanone, 5-methyl-	-	-	-	363.28 ± 41.66	158.16 ± 51.53	-	-		
5-Hepten-2-one, 6-methyl-	-	-	-	-	-	-	-		
3-Octanone	-	-	-	320.10 ± 11.98	162.07 ± 18.93	453.04 ± 26.82	-		
Cyclohexanone, 2,2,6-trimethyl-	-	-	-	-	-	-	-		
Isophorone	-	-	-	-	-	-	-		
Ionone	-	-	-	-	-	-	-		
2-Buten-1-one, 1-(2,6,6-trimethyl-1,3-cyclohexadien-1-yl)-, (E)-	38.98 ± 1.38			604.16 ± 45.61	170.23 ± 12.67	80.19 ± 7.00	70.48 ± 3.40		
1(2H)-Naphthalenone, 3,4-dihydro-3,3,6,8-tetramethyl-	-	-	-	-	-	19.66 ± 7.90	-		
3-Buten-2-ol, 2-methyl-	-	-	-	-	-	-	-		
1-Butanol	-	-	-	-	-	-	-		
Cyclohexanol, 4-methyl-, trans-	-	-	623.17 ± 32.32	-	-	-	-		
5-Hexen-2-ol, 5-methyl-	-	-	619.6 ± 20.15	-	-	-	-		
Cyclohexanol, 4-methyl-, cis-	-	-	-	-	-	-	-		
Cyclohexanol, 4-methyl-	-	-	-	-	-	-	-		
1-Butanol, 2-methyl-	-	-	-	-	-	-	-		
1-Butanol, 3-methyl-	-	-	-	-	-	-	-		
1-Pentanol	-	-	-	-	197.98 ± 30.88	-	-		
2-Penten-1-ol, (Z)-	-	-	-	-	-	189.20 ± 12.43	-		
3-Hexen-1-ol, (E)-	-	-	-	-	-	-	-		
4-Hexen-1-ol, (E)-	-	-	14,101.03 ± 380.22	-	-	-	17,745.86 ± 201.92		
2-Hexen-1-ol, (E)-	-	-	3066.92 ± 60.99	11,651.56 ± 1894.27	4201.80 ± 325.14	10,502.51 ± 85.34	-		
1-Hexanol	4309.79 ± 27.21	5444.43 ± 26.38	3453.93 ± 107.05	6470.01 ± 11,162.58	11,313.03 ± 59.47	18.81 ± 1.28	-		
1-Butanol, 2-methyl-, acetate	-	-	-	-	-	-	-		
Ethanol, 2-butoxy-	-	-	-	-	-	-	-		
4-Penten-1-ol, propanoate	-	-	-	-	-	-	-		
3-Hexanol, 3-ethyl-	-	-	-	-	-	-	-		
3-Heptanol	13.44 ± 1.08	29.72 ± 8.79	-	40.84 ± 2.58	30.45 ± 3.21	104.44 ± 2.04	-		
1-Octen-3-ol	18.80 ± 5.65	23.82 ± 4.47	23.67 ± 2.88	24.18 ± 0.79	34.22 ± 5.86	108.08 ± 7.84	-		
1-Hexanol, 2-ethyl-	1412.61 ± 53.79	3100.97 ± 232.15	2709.80 ± 54.59	9212.51 ± 152.84	7518.37 ± 144.20	4265.41 ± 113.67	-		
1-Octanol	29.59 ± 2.62	-	-	72.66 ± 4.47	33.14 ± 11.07	-	-		
Linalool	448.47 ± 9.46	1515.31 ± 26.09	283.19 ± 9.68	2797.09 ± 265.65	232.60 ± 24.10	-	1168.29 ± 48.98		
1,5,7-Octatrien-3-ol, 3,7-dimethyl-	-	-	104.60 ± 1.29	-	-	-	-		
Hotrienol	-	-	-	434.46 ± 45.54	-	-	-		
trans-Ocimenol	-	-	-	-	-	-	-		
L-.alpha.-terpineol	-	304.34 ± 3.84	-	-	-	-	283.64 ± 9.16		
alpha.-terpineol	-	298.73 ± 3.42	-	429.92 ± 42.63	-	-	-		
3-Buten-1-ol, 3-methyl-	-	-	-	82.20 ± 24.49	-	-	-		
2,6-Octadien-1-ol, 3,7-dimethyl-, (Z)-	-	-	-	-	-	-	-		
Pentanoic acid, 3-methyl-4-oxo-	-	-	-	-	-	-	-		
Butanoic acid	-	-	-	-	-	-	-		
1-Hexyne, 5-methyl-	-	-	-	-	-	-	-		
4-Octenoic acid, ethyl ether	-	-	-	-	-	-	-		
Phenol, 4-(2-propenyl)-	-	-	-	-	-	-	-		
Methyleugenol	-	-	-	-	-	-	-		

Note: - indicates not detected.

**Table 3:** Eigenvalues and variance contribution rates of principal components.

Principal Components	Initial Eigenvalue		
	Eigenvalues	Rate of Variance/%	Rate of Cumulative Variance/%
1	22.115	15.910	15.910
2	14.405	10.363	26.274
3	11.353	8.168	34.442
4	9.167	6.595	41.036
5	6.836	4.918	45.954
6	6.301	4.533	50.487
7	5.725	4.118	54.606
8	5.624	4.046	58.652
9	5.038	3.624	62.276
10	4.856	3.494	65.770
11	4.382	3.152	68.922
12	4.089	2.942	71.864
13	3.821	2.749	74.613
14	3.393	2.441	77.054
15	3.270	2.353	79.407
16	3.128	2.250	81.657

**Table 4:** Key aroma components and OAVs in 38 plum germplasm resources.

Key Aroma Components	Odor Threshold [20,21,22,23,24,25,26] (μg/kg)	OAVs																		
		1	2	3	4	5	6	7	8	9	10	11	12	13	14	15	16	17	18	19
2-Hexenal, (E)-	17	16.11	22.48	20.82	20.94	1.79	1.38	4.18	450.78	31.85	40.40	36.11	33.91	3.16	17.72	12.28	5.56	5.46	27.63	3.12
2-Hexenal	30	599.11	945.01	480.90	420.35	144.56	281.14	157.98	83.10	1273.47	1356.64	700.76	1404.74	104.561	311.38	397.83	117.95	305.55	439.47	66.74
1-Cyclohexene-1-carboxaldehyde, 2,6,6-trimethyl-	3	-	44.180	-	10.43	-	-	-	-	-	-	-	-	-	252.37	54.27	-	-	-	-
Octanal	0.7	-	-	-	-	-	-	120.06	-	-	-	-	-	-	-	-	-	-	-	-
Ethyl acetate	1000	-	-	-	-	-	3.62	-	-	-	-	-	-	-	-	-	-	-	-	-
Formic acid, butyl ester	370						4.23	-	-	-	-	-	-	-	-	-	-	-	-	-
Acetic acid, butyl ester	100	-	-	-	-	-	345.38	-	28.25	-	16.68	66.75	-	5.52	-	-	15.51	30.88	37.51	41.88
Pentanoic acid, ethyl ester	3	-	-	-	-	-	-	-	3.92	-	-	-	-	-	-	-	-	-	-	-
Acetic acid, pentyl ester	430	-	-	0.15	-	15.45	4.39	-	-	-	-	-	-	-	-	-	-	-	-	0.36
Hexanoic acid, methyl ester	84	-	-	-	-	7.20	1.48	-	-	-	-	-	-	-	-	-	-	-	-	-
Hexanoic acid, ethyl ester	1	-	-	-	-	-	-	143.91	-	-	-	-	-	-	-	-	-	-	-	-
3-Hexen-1-ol, acetate, (Z)-	16	2.29	359.44	1031.73	167.28	972.56	952.71	15.97	657.88	47.99	495.01	237.38	1.12	68.53	248.11	511.77	344.87	24.58	93.40	35.14
Butyl 2-methylbutanoate	80	-	-	-	-	15.35	8.54	-	-	-	-	-	-	-	-	-	-	-	-	-
Linalyl acetate	100	-	-	-	-	-	-	-	-	-	-	-	-	-	10.16	-	-	-	-	-
1-Octen-3-one	0.1	-	-	-	199.28	-	-	-	-	-	-	-	-	-	-	-	-	-	-	-
1-Butanol	500	-	-	-	-	6.29	-	-	-	-	-	-	-	-	-	-	-	-	-	-
1-Butanol, 2-methyl-	1000	-	-	0.06	-	2.79	-	-	-	0.16	0.10	-	-	-	-	-	-	0.07	-	0.06
1-Butanol, 3-methyl-	160	-	-	-	-	-	-	-	-	-	7.18	-	-	-	-	-	2.67	-	-	-
1-Octanol	190	-	-	0.30	-	1.72	-	-	-	-	-	-	0.08	-	-	-	0.68	-	-	-
Hotrienol	110	-	-	-	-	-	-	-	-	-	-	-	-	-	-	-	-	-	-	-
L-.alpha.-terpineol	330	-	-	19.38	8.34	-	-	-	0.52	0.83	-	-	1.96	-	-	0.46	0.93	-	-	-
alpha.-terpineol	12.5	-	225.37	-	-	-	-	-	21.91	19.33	8.75	-	-	-	12.78	-	-	-	-	-
2,6-Octadien-1-ol, 3,7-dimethyl-, (Z)-	49	-	1.24	-	-	-	-	-	-	-	-	0.72	-	-	-	-	-	-	-	-
Butanoic acid	90	-	-	-	-	1.71	-	-	-	-	-	-	-	-	-	-	-	-	-	-
Key Aroma Components	Odor Threshold (μg/kg)	OAVs																		
		20	21	22	23	24	25	26	27	28	29	30	31	32	33	34	35	36	37	38
2-Hexenal, (E)-	17	1.02	1.00	-	64.39	9.39	16.69	11.65	5.10	-	36.78	19.34	10.83	12.71	31.99	14.82	92.73	36.02	119.56	0.52
2-Hexenal	30	64.55	96.56	248.10	1944.31	126.58	650.27	171.32	84.51	243.68	870.15	450.21	136.72	204.56	630.88	250.73	1422.36	1372.15	4542.16	-
1-Cyclohexene-1-carboxaldehyde, 2,6,6-trimethyl-	3	-	39.12	-	-	-	54.63	-	57.55	-	-	-	-	97.35	61.94	-	-	-	-	-
Octanal	0.7	-	-	-	-	-	-	-	-	-	-	-	-	-	-	-	-	-	-	-
Ethyl acetate	1000	-	-	-	-	-	-	-	-	-	-	-	-	-	-	-	-	-	-	-
Formic acid, butyl ester	370	-	-	-	-	-	-	-	-	-	-	-	-	-	-	-	-	-	-	-
Acetic acid, butyl ester	100	1649.51	631.54	165.40	23.24	-	-	-	-	57.17	101.85	313.15	-	15.45	13.27	6.59	-	-	-	48.87
Pentanoic acid, ethyl ester	3	-	-	-	-	-	-	-	-	-	-	-	-	-	-	-	-	-	-	-
Acetic acid, pentyl ester	430	9.26	3.94	0.17	0.09	-	-	-	-	0.05	0.07	0.28	-	-	-	-	-	-	-	0.11
Hexanoic acid, methyl ester	84	-	-	-	-	-	-	-	-	-	-	-	-	-	-	-	-	-	-	-
Hexanoic acid, ethyl ester	1	-	-	4036.88	-	-	-	-	-	3049.16	-	-	-	-	-	-	-	-	-	-
3-Hexen-1-ol, acetate, (Z)-	16	461.58	154.72	269.17	25.90	3.40	0.00	0.00	0.00	169.15	390.92	768.54	0.00	191.08	241.71	255.22	3313.99	0.00	0.00	233.27
Butyl 2-methylbutanoate	80	0.37	2.86	-	-	-	-	-	-	-	-	-	-	-	-	-	-	-	-	-
Linalyl acetate	100	-	-	-	-	-	-	-	-	-	-	-	-	-	-	-	-	-	-	-
1-Octen-3-one	0.1	-	-	-	-	-	-	-	-	-	-	-	-	-	-	-	-	-	-	-
1-Butanol	500	1.16	10.96	-	-	-	-	-	-	-	-	0.89	-	-	-	-	-	-	-	-
1-Butanol, 2-methyl-	1000	1.32	4.59	0.09	-	-	-	-	-	0.18	-	0.04	-	-	-	-	0.30	0.22	0.05	0.11
1-Butanol, 3-methyl-	160	1.09	2.10	5.11	-	-	-	-	-	-	-	-	-	-	-	-	-	-	-	-
1-Octanol	190	0.31	0.29	0.14	0.21	-	-	-	-	0.16	0.26	0.22	-	-	-	-	0.38	0.17	-	-
Hotrienol	110	-	-	-	-	-	-	-	-	-	-	-	-	-	-	-	3.95	-	-	-
L-.alpha.-terpineol	330	-	-	-	-	-	-	-	-	-	-	-	-	0.28	0.92	-	-	-	-	0.86
alpha.-terpineol	12.5	-	-	-	-	-	-	-	-	-	-	-	-	-	23.90	-	34.39	-	-	-
2,6-Octadien-1-ol, 3,7-dimethyl-, (Z)-	49	-	-	-	-	-	-	-	-	-	-	-	-	-	-	-	-	-	-	-
Butanoic acid	90	-	-	-	-	-	-	-	-	-	-	-	-	-	-	-	-	-	-	-

Note: - indicates not detected or not calculated.

**Table 5:** Key aroma components of 38 plum germplasm.

Specific Aroma Components of Key Aroma Components (OAV ≥ 10)	Aroma Character	Germplasm
(E)-2-Hexenal	Green, aldehyde, fruity, spicy, and fatty. Strong and sharp before dilution, and a pleasant green, leafy and fruity aroma after dilution	Hongshou, Lixingzazhong, Zaoli, Jizaofeng, Gaida, Jihong, Taiyang, Taoxingli, Aitianli, Huangganhe, Jisheng, Dahongpao, Dahongpao, Qiuji, XY, XY 7-2, Guofeng 2, Qiulizi, Konglongdan, Angeleno, Wanhuang, Liwang, Friar Plum, Great Rosa, Richard Early
2-Hexenal	Leafy	Hongshou, Lixingzazhong, Zaoli, Jizaofeng, Dadanguo, Xiangjiaoli, Hongxing, Gaida, Jihong, Taiyang, Taoxingli, Aitianli, Zhushali, Huangganhe, Jisheng, Hongxinli, 10-27, Dahongpao, Hongbayi, Jinxixiangjiaoli, Heibali, Suili 3, Qiuji, 98-1-6, XY, XY 7-2, XY 7-1, Xingguang 2, Guofeng 2, Qiulizi, Konglongdan, Angeleno, Wanhuang, Liwang, Friar Plum, Great Rosa, Richard Early
2,6,6-trimethyl-1-Cyclohexene-1-carboxaldehyde	Tropical fruity aroma, herbaceous	Lixingzazhong, Jizaofeng, Huangganhe, Jisheng, Heibali, XY, XY 7-1, Angeleno, Wanhuang
Octanal	Extremely diluted with aromas similar to rose and orange peel	Hongxing
Acetic acid butyl ester	Fruity	Xiangjiaoli, Gaida, Taiyang, Taoxingli, Hongxinli, 10-27, Dahongpao, Hongbayi, Jinxixiangjiaoli, Heibali, Suili 3, Qiuji, Xingguang 2, Guofeng 2, Qiulizi, Angeleno, Wanhuang, Fortune
Acetic acid pentyl ester	Fruity and sweetness	Dadanguo
Hexanoic acid ethyl ester	Pineapple	Hongxing, Suili 3, Xingguang 2
(Z)-3-Hexen-1-ol acetate	Strong banana	Hongshou, Lixingzazhong, Zaoli, Jizaofeng, Dadanguo, Xiangjiaoli, Hongxing, Gaida, Jihong, Taiyang, Taoxingli, Aitianli, Zhushali, Huangganhe, Jisheng, Hongxinli, 10-27, Dahongpao, Hongbayi, Jinxixiangjiaoli, Heibali, Suili 3, Qiuji, 98-1-6, Xingguang 2, Guofeng 2, Qiulizi, Angeleno, Wanhuang, Liwang, Friar Plum, Fortune
Butyl 2-methylbutanoate	/	Dadanguo
Linalyl acetate	/	Huangganhe
1-Octen-3-one	Herbs, mushrooms, soil	Jizaofeng
1-Butanol	Sweet, vinegar, whiskey	Heibali
L-.alpha.-terpineol	With a fragrant scent resembling that of a sea paulownia flower, sweet lilac, and lily of the valley aromas	Zaoli
alpha.-terpineol	Has a lilac-like aroma	Lixingzazhong, Gaida, Jihong, Huangganhe, Wanhuang, Friar Plum

Note: Most of the characteristic aroma components were searched from http://www.thegoodscentscompany.com/index.html (accessed on 14 August 2024); ‘/’ indicates that the substance has not been detected or found.

## Data Availability

The original contributions presented in the study are included in the article, further inquiries can be directed to the corresponding author.

## Abbreviations

HS-SPME-GC-MS	Headspace solid-phase microextraction gas chromatography–mass spectrometry
OPLS-DA	Orthogonal partial least squares discriminant analysis
VIP	Variable importance projection
PCA	Principal component analysis
